# Diversification of Transposable Elements in Arthropods and Its Impact on Genome Evolution

**DOI:** 10.3390/genes10050338

**Published:** 2019-05-06

**Authors:** Changcheng Wu, Jian Lu

**Affiliations:** State Key Laboratory of Protein and Plant Gene Research, Center for Bioinformatics, School of Life Sciences, Peking University, Beijing 100871, China; ccwu@pku.edu.cn

**Keywords:** transposable elements, evolution, arthropods, genome size, horizontal transfer, database

## Abstract

Transposable elements (TEs) are ubiquitous in arthropods. However, analyses of large-scale and long-term coevolution between TEs and host genomes remain scarce in arthropods. Here, we choose 14 representative *Arthropoda* species from eight orders spanning more than 500 million years of evolution. By developing an unbiased TE annotation pipeline, we obtained 87 to 2266 TE reference sequences in a species, which is a considerable improvement compared to the reference TEs previously annotated in Repbase. We find that TE loads are diversified among species and were previously underestimated. The highly species- and time-specific expansions and contractions, and intraspecific sequence diversification are the leading driver of long terminal repeat (LTR) dynamics in Lepidoptera. Terminal inverted repeats (TIRs) proliferated substantially in five species with large genomes. A phylogenetic comparison reveals that the loads of multiple TE subfamilies are positively correlated with genome sizes. We also identified a few horizontally transferred TE candidates across nine species. In addition, we set up the Arthropod Transposable Elements database (ArTEdb) to provide TE references and annotations. Collectively, our results provide high-quality TE references and uncover that TE loads and expansion histories vary greatly among arthropods, which implies that TEs are an important driving force shaping the evolution of genomes through gain and loss.

## 1. Introduction

Transposable elements (TEs) are DNA sequences that can jump in host genomes [1]. TEs are widespread in eukaryotic organisms and occupy more than 45% of the human genome [2]. Previous studies showed that TEs mainly adopt two mechanisms in replication: “copy and paste” and “cut and paste” [3]. The first class of TEs are mainly retrotransposable elements that require RNA intermediates, and the second class of TEs are mainly DNA transposons (terminal inverted repeats or TIRs). As TE translocation might cause genomic instabilities or waste energy of the host organisms, TEs used to be regarded as “junk DNA” [4].

The genomes of many arthropods have been sequenced in the past decades, which suggests the contents of TEs are highly variable in *Arthropoda* [5,6,7,8,9,10,11,12,13,14,15,16,17,18]. For instance, *Locusta migratoria* has a huge genome, which is larger than 6.5 Gb [17], while *Tetranychus urticae* has a much smaller genome which is less than 0.1 Gb [9]. As the number of genes does not differ significantly between these two species [9,17], the 65-fold genome size variation might be mainly due to the rapid evolution of TEs. Consistently, a very recent study surveyed TEs in 62 insects and 11 non-insect outgroup species and found TE contents vary considerably in insect genomes, suggesting the variation in genome size is shaped by the expansion and contraction of TEs in arthropods [19]. TE gain and loss is one of the major drivers of genome size changes, as previously shown in mammals [20], avians [21] and *Drosophila* [22].

Nevertheless, several unaddressed gaps remain in our understanding of the evolutionary dynamics of TEs and their impact on the evolution of arthropod genomes. First, most of the identified TE sequences in arthropods are based on the reference sequences in the Repbase database, and many species-specific TEs that are under-represented in Repbase are not well recovered. Second, many TE annotation programs such as RepeatModeler does not consider the global structure of a TE, which might identify a partial but not the full-length sequence of a TE. Third, the expansion and contraction dynamics for most TE families or subfamilies in arthropods are still not well understood. Forth, it remains unclear how frequently horizontal transfer of TEs (HTTs) occur in arthropods. Arthropods have tremendously diversified phenotypes and abundant genomic resources. Answers to the above questions might help understand the roles of TEs during the diversification of arthropods.

We explored the evolutionary dynamics of TEs in fourteen representative *Arthropoda* species spanning eight orders. We first built a high-quality TE reference library for each species by combining sequence homology searching and *de novo* TE identification. Then, we explored TE expansion and contraction in these arthropod species based on the phylogenetic tree. We found that frequent gains and losses, sequence diversification, and HTTs jointly contributed to TE load diversity in arthropods. Finally, we report the database ArTEdb (http://db.cbi.pku.edu.cn/arte), which incorporates the sequences and annotations of TEs identified in this study. The resources provided by this study will benefit future TE studies in arthropods.

## 2. Materials and Methods

### 2.1. Transposable Elements Reference Construction

The genome sequences were downloaded from the NCBI, FlyBase, and SilkBase databases (Table S1). The published TEs were downloaded from Repbase (v23.02) [23]. LTRs have several structural features, including target site repeats, long terminal repeats, primer binding sites (PBSs), polypurine tract (PPT) and multiple open reading frames (ORFs). The ORFs in long terminal repeats (LTRs) encode functional domains such as reverse transcriptase (RT), integrase (IN), and RNase H (RH). Reverse transcription of LTR requires tRNA primer that pairs with the PBS. Therefore, the domain profiles and tRNAs will help to identify and classify LTRs better. LTR domain profiles were downloaded from GypsyDB (www.gydb.org) [24]. The tRNAs were annotated using tRNAScan-SE (-G) [25]. Only high-quality tRNAs (score > 40) with clear anticodons were kept and used in the LTR annotation. TE reference libraries were built using both homology-based and *de novo* methods.

### 2.2. Identification and Annotation of Transposable Elements 

Two *de novo* tools were used to identify full-length LTR candidates initially. LTR_Finder uses tRNAs and Pfam domain profiles (-w 2 -l 100 -L 1000 -D 12000 -d 2000) [26], and LTRharvest (-seed 80 -minlenltr 100 -maxlenltr 1000 -mindistltr 2000 -maxdistltr 12000 -overlaps no -similar 80 -mintsd4 -maxtsd 20 -longoutput) is one module of GenomicTools [27]. The tRNAs and Pfam domains profiles are used for identifying PBSs and enzyme domains respectively. LTRdigest (-pptlen 10 30 -pbsoffset 0 3) [28] was applied to refine the identifications using both tRNAs and LTR domain profiles downloaded from GypsyDB. Only LTR candidates with at least one of the five LTR domains (GAG, AP, INT, RT, and RH) were kept. All identified LTR candidates were combined and clustered with the UCLUST (id = 0.9) algorithm [29]. For TEs in each cluster, CLUSTALW2 [30] was applied to perform multiple alignments, and the cons (EMBOSS) tool was used to build consensus sequences. Singletons (only one TE in a cluster) having at least four of the five LTR domains were kept. Both singletons and consensus sequences were masked by RepeatMasker [31] with the built-in libraries of corresponding species. Sequences that overlapped with RepeatMasker libraries (more than 80% of the queries were masked) were collapsed.

Besides the LTR specific annotation programs, we also employed RepeatModeler (www.repeatmasker.org/RepeatModeler) pipeline with the default parameters to identify TEs in each species. Consensus sequences aligned to known protein-coding genes of *Drosophila melanogaster* were removed. Moreover, the remaining sequences that overlapped with previous annotations of Repbase or LTRs were collapsed. All annotated TE sequences by RepeatModeler were combined, and their classes and subfamilies were further determined based on both sequence similarity to known TEs (Repbase) or TE-specific domains (Pfam_v27 and GypsyDB) by PASTEClassifier [32] and *de novo* classifications by TEclass [33].

For each species, the TEs identified by the LTR specific tools and RepeatModeler were combined. USEARCH [29] was used to obtain the nonredundant TE libraries for each species. All the TE references for each species can be downloaded from the ArTEdb database (http://db.cbi.pku.edu.cn/arte). For each TE reference annotated in this study, we denote it with the TE class followed by the first three letters of the genus and the first three letters of the species name. For example, the homologous sequence of *Gypsy-1_DSim* in *Drosophila melanogaster* is *Gypsy-1_DroMel* in the ArTEdb database.

### 2.3. Transposable Element Loads and Expansion Analyses

Genomes were masked by RepeatMasker using TE libraries defined in this study. TE loads in each species were calculated using the script ONE_CODE_TO_FIND_THEM_ALL.PL [34]. The Kimura 2-Parameter divergence of TEs was calculated using the RepeatMasker built-in tool calcDivergenceFromAlign.pl, and the distributions of divergence were plotted using ECharts (www.echarts.baidu.com).

### 2.4. Reconstructing the Phylogenetic Tree

BUSCO [35] was adopted with the insect core genes to annotate single-copy orthologous genes in the fourteen species. Only single-copy genes with intact ORFs were kept. Orthologous protein multiple alignments generated by T-COFFEE [36] were then transformed into codon alignments using RevTrans [37]. A preliminary phylogeny tree for the selected species was firstly reconstructed by MEGA [38] based on the concatenated protein alignments of orthologous genes. Sites with more than 50% gaps were removed from the concatenated alignment, and the phylogeny was built using Maximum-likelihood algorithm with the JTT matrix. The topological position of *L. migratoria* was manually curated based on a previous study [39]. All the codon alignments were concatenated, and CODEML from PAML [40] was used to calculate the *dN* with the free model (runmode = 0; model = 1). The *dN* values of the concatenated sequences were then set as the branch length of the phylogenetic tree. The tree is provided in the Supplementary File S1.

### 2.5. Time-Calibrated Phylogeny

The non-parametric tool r8s [41] was used to transform the branch lengths into millions of years. Fossil evidence shows that the divergence time between *Bombus terrestris* and *Apis mellifera* is 23 to 28.4 million of years ago (Ma) [39], and the age of the root node was set as 550 to 580 Ma [39,42].

### 2.6. Fitting Multiple Phylogenetic Comparative Models

Both the TE loads and genomes sizes (gaps excluded) were transformed in natural log(Ln)grams. Their phylogenetic signals were estimated using the phylosig (method = “lambda”) function from phytools [43]. The adequacy of four standard phylogenetic comparative models were tested using the fitContinuous function of Geiger [44] in R. These models are Brownian motion (BM) [45], Ornstein-Uhlenbeck (OU) [46], Early-burst (EB; also named as Accelerating-Decelerating (ACDC)) [47], and white noise (non-phylogenetic and normal distribution). The AICc (corrected Akaike information criterion for small sample size) values of these models were evaluated, and the results suggest that the BM model was the most suitable model. Therefore, the BM model was used in the next phylogenetically independent contrasts and ancestral state reconstruction of TE loads.

### 2.7. Phylogenetically Independent Contrasts

The phylogenetically independent contrasts (PIC) of TE loads and host genome sizes (gaps excluded) were calculated using the pic function of ape [48] in R. The TE loads of all subfamilies were added to the total TE loads. For the four main classes (TIR, LTR, LINE, and SINE), the TE loads of all subfamilies belong to them were added together. Subfamilies that appeared in more than seven species were preserved for the additional subfamily-level PIC analyses. Both the TE loads and genome sizes were in natural log(Ln)grams. The correlation between TE loads and host genome sizes were calculated using Pearson’s product-moment correlation test in R. The *P* values of these subfamilies were corrected using the Holm-Bonferroni correction [49].

### 2.8. Ancestral State Reconstruction of Transposable Element Loads

The TE loads that are in natural log(Ln)grams were fitted to the Brownian motion model. The TE load states of the ancestor nodes were inferred by the phytools [43] using the maximum-likelihood analysis method [50]. The fastAnc function from phytools was used to reconstruct the ancestral state as previously reported [51]. Similar to the method used in a previous study [51], the TE load change ratio was defined as the TE load of offspring node relative to its ancestral node. The phylogenetic tree with TE load change ratios was plotted using ReproPhylo [52].

### 2.9. Identifying Shifts of Transposable Element Loads Change Rates in the Phylogeny

BAMM (Bayesian analysis of macroevolutionary mixtures) [53,54,55] was used to identify shifts of TE loads evolutionary rates in the time-calibrated phylogeny. The betaInitPrior and betaShiftPrior parameters were estimated by the setBAMMpriors function of BAMMtools v2.1.6 [56], and 1,000,000 generations of MCMC (Markov chain Monte Carlo) sampling were conducted. The outputs of BAMM were then post-processed by BAMMtools, and the evolutionary rates of TE loads and the best shift configuration were extracted and highlighted in the phylorate plots for four TE classes and three subfamilies (TIR/Mariner, LTR/Gypsy, and LINE/Jockey).

### 2.10. Transposable Element Protein Annotation

Proteins of TEs were annotated with the homology-based method. TE proteins extracted from Repbase were aligned to annotated TEs using the tblastn program [57]. For each TE, the aligned protein with the smallest E-value was selected. All preserved query-target pairs were then realigned using exonerate (--model protein2genome:bestfit) to annotated proteins [58].

### 2.11. The Phylogenetic Analyses of LTRs in LEPIDOPTERA

The *pol*-encoded proteins of LTRs were first aligned through T-COFFEE [36]. For the alignments of *Gypsy* and *Pao*, aligned sites covered by less than 70% of all TE sequences were removed. FastTree was applied to build the phylogenetic trees for *Copia*, *Gypsy,* and *Pao* [59]. The phylogenetic trees were plotted using FigTree (https://github.com/rambaut/figtree).

### 2.12. Horizontal Transposable Element Transfer

Due to the large evolutionary distances among the fourteen-selected species, we adopted the genome-wide amino acid distance instead of *dS* (synonymous substitutions per synonymous site) as the cutoff for identifying HTT. Protein sequences of single-copy orthologous genes that were annotated using BUSCO with core genes of Insecta lineage were aligned using CLUSTALW2 [30] between each pair of species. The amino acid distances were calculated using PAML (aaRatefile = jones.dat) and sorted in ascending order. Since the number of orthologous genes between every two arthropods might be larger than 5000 [60], and our BUSCO analyses might have captured the most conserved ones, therefore, we set the genome-wide cutoff as the 100th minimum amino acid distance between two species, which represents the top ~2% of the total orthologous genes (Table S3). TE proteins were aligned between each pair of fourteen-selected species, and only reciprocal best hits were kept. The amino acid distances of the aligned TEs were calculated as described above. TE pairs with lower amino acid distances than genome-wide cutoffs were selected as HTT candidates.

### 2.13. Analysis of Transposable Elements Distribution in Arthropods

Genomes of 126 extra insect species were downloaded from the InsectBase (http://insect-genome.com). For each pair of HTT candidates, we aligned them to genomes of all 140 arthropods using the fasta36 (-E 1e-5) program of FASTA [61]. All hits that are longer than 80 bp and have more than 80% similarity were kept.

### 2.14. The ArTEdb Database

The ArTEdb was written in HTML and PHP and hosted in Apache. All TE information was organized using MySQL in the background. Both gene and TE annotations were embedded in JBrowse. Alignment|Blast uses NCBI BLAST (v2.7.1+) [57] in the background with TE references from 14 arthropods as databases. The alignment results are post processed by xmlBLASTparser (www.github.com/AshokHub/xmlBLASTparser) for visualization. Alignment|RepeatMasker runs RepeatMasker in the background.

## 3. Results

### 3.1. Construction of Transposable Element References

**Figure 1:**
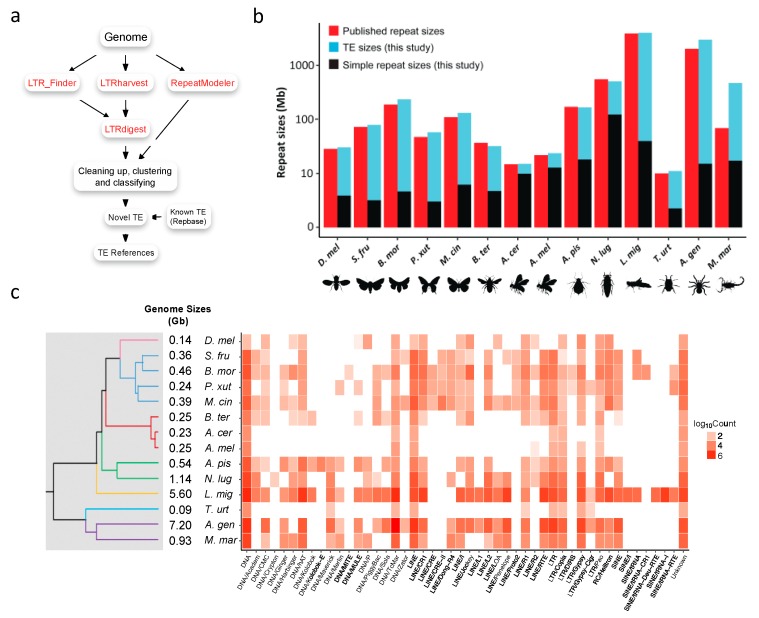
Annotating TEs in arthropods. (**a**) The TE reference construction workflow (see details in Materials and Methods). (**b**) The repeat sizes of fourteen arthropods. Published repeat sizes were adapted from the original genome sequencing studies (Table S1). The repeat sizes (TE and simple repeat) of this study were evaluated using the TE libraries defined in this study. The published repeat size of *B. mor* was adapted from [11]. *D. mel*, *Drosophila melanogaster*; *B. mor*, *Bombyx mori*; *M. cin*, *Melitaea cinxia*; *P. xut*, *Papilio xuthus*; *S. fru*, *Spodoptera frugiperda*; *A. cer*, *Apis cerana*; *A. mel*, *Apis mellifera*; *B. ter*, *Bombus terrestris*; *N. lug*, *Nilaparvata lugens*; *A. pis*, *Acyrthosiphon pisum*; *L. mig*, *Locusta migratoria*; *A. gen, Acanthoscurria geniculata*; *M. mar*, *Mesobuthus martensii*; *T. urt*, *Tetranychus urticae*. (**c**) The landscape of TE loads in arthropods.

Although Repbase [23] provides TE annotations for many arthropods, high-quality TE references are available for only a small subset of the 14 species investigated in this study (Table 1). To obtain high-quality and unbiased TE references, we systematically annotated TEs from genomes of all fourteen species using the pipeline described in Figure 1a. We obtained 87 to 2266 TE reference sequences in a species, which is a considerable improvement compared to the reference TEs previously annotated in Repbase (Table 1). Notably, much higher numbers of reference TEs were identified in the species that have large genomes, for instance, *Acanthoscurria geniculata* and *Locusta migratoria*, than the species that have small genomes (Table 1). Eight of the fourteen species had few TEs (≤10) annotated in Repbase, while hundreds of extra reference TEs were identified in this study (Table 1).

The new high-quality TE references inspired us to ask whether TE contents were underestimated in previous studies. To answer this question, we evaluated the repeat sizes and TE contents in the selected species with the new TE references (Figure 1b; Table S1). The results show that repeat sizes were previously underestimated in 11 of the 14 selected species, especially those with few TE references annotated in Repbase. For instance, no TE reference had been reported in *A. geniculata* (the original genome sequencing study reported that approximately 60% of the genome excluding N/X runs was composed of TEs, but TE references were not available [16]), and the initial repeat masking by RepeatMasker with the built-in *Arthropoda* library showed that the TE content was only 4.5%. However, 57.17% of the *A. geniculata* genome was masked using the new TE references, which is very close to the number obtained in the original study. In addition, we masked its full genome and identified extra 950 Mb TEs. Another striking species is *Mesobuthus martensii*. Because of the limited number of annotated TEs in Repbase, the TE size of this species was previously underestimated to be 35 Mb (3.1%) [7]. Here, we identified 1400 TE references in *M. martensii* and revealed that approximately half (51.03%; 455 Mb TEs and 17 Mb simple repeats) of its genome consisted of repeats (Figure 1b).

Even for *D. melanogaster*, whose TEs are well annotated in Repbase, our annotation results still identified another 87 TE subfamilies (8 LTRs and 79 non-LTRs). For example, although *Gypsy-1_DSim* is a well known LTR in *Drosophila simulans*, no homologs of this TE have been reported in *D. melanogaster*. Here we found that the *Gypsy-1_DSim* TE subfamily has 117 copies in the Y chromosome and another 252 copies (at least six full-length ones) in the other five chromosomes (2L/2R/3L/3R/X) of *D. melanogaster* (these TEs were named as *Gypsy-1_DroMel* in our ArTEdb database). Interestingly, the full-length *Gypsy-1_DroMel* was successfully identified by LTR_Finder and LTRharvest, while RepeatModeler only identified a partial sequence of this TE in *D. melanogaster* (Figure S1), which suggests that the approach we employ by combining multiple annotation tools is more powerful than using RepeatModeler alone.

In summary, we annotated TEs for fourteen *Arthropoda* species with the unbiased pipeline and identified many more novel TEs. These high-quality TE libraries might contribute to a better understanding of the evolution and genome contributions of TEs in arthropods.

### 3.2. Transposable Element Loads Vary Greatly in Arthropods

The huge difference in genome sizes among fourteen arthropods raises the question of how much TEs contribute to their hosts. To answer this question, we masked the genomes of arthropods using RepeatMasker with the new TE references and calculated the TE loads of each species. Hereafter, the TE load is defined as the copy number of TEs in the genome. The loads of multiple TE subfamilies from four main TE classes (TIR, LINE, LTR, and SINE) were summed. Although three (TIR, LINE, and LTR) of the classes existed in all fourteen species, the TE loads varied greatly among species (Figure 1c). The diversity of TEs in arthropods was also reported in a recent study [19]. Next, we will focus on several remarkable insights to show how TEs contribute to genome evolution.

#### 3.2.1. Transposable Element Loss is Prevalent in *Hymenoptera*

The three *Hymenoptera* species, namely, *Apis mellifera*, *Apis cerana,* and *Bombus terrestris,* have the closest evolutionary relationship among the fourteen species. The TE load analysis reveals that these three species have fewer TEs than other species except for *T. urticae* (Figure 1b–c). Especially in the first two species, the repeat sequences account for less than 10% of the host genome, and the real TE content is even lower than 5%. Although we observed several LTRs and LINEs, none of them had coding potential. Only a few *Mariner* (DNA/TcMar) TEs have intact ORFs, consisting with the previous study [8]. Comparing TE loads in *A. mellifera* and *A. cerana* with those in the remaining species, we observed that the TE loss occurred rapidly in *A. mellifera* and *A. cerana*. These two evolutionarily close species had a slight difference in their TE loads, and both the subfamily numbers and the TE loads were significantly reduced (Figure 1c). However, in *B. terrestris*, most TE subfamilies could still be observed, and repeats occupied approximately 13.5% (simple repeat < 2%) of its genome. These results reveal that the extent of TE loss is highly diverse among *Hymenoptera* and somewhat weak in *B. terrestris*.

#### 3.2.2. Recent LTR Expansion in *T. urticae*

**Figure 2:**
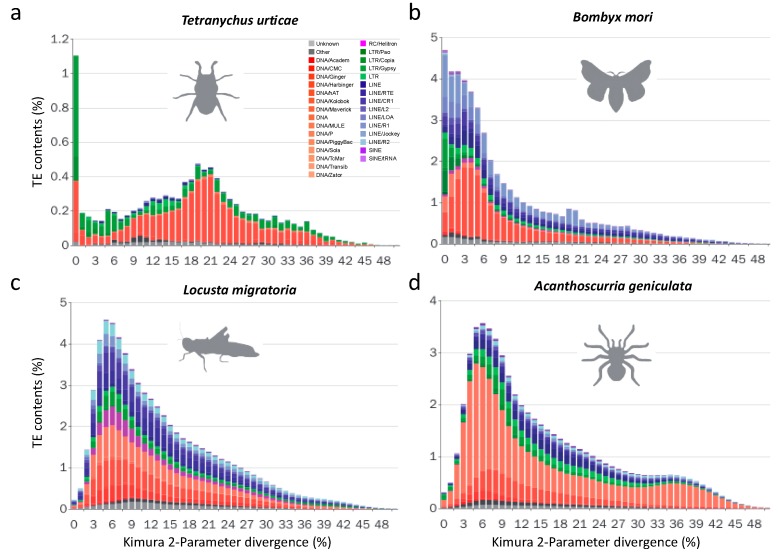
Sequence divergence distribution of TEs. (**a**–**d**) Distribution of sequence divergence of multiple TE subfamilies in four species. The *y-axis* shows the percentage of the host genomes that is annotated as TEs (TE contents). The *x-axis* shows Kimura 2-Parameter sequence divergence between individual TE copies and consensus references. Unknown, unclassified TEs; Other, Retro and Retroposon.

In addition to these two *Hymenoptera* species (*A. mellifera* and *A. cerana*), *T. urticae,* which has the smallest genome (90 Mb) among the fourteen species, also has fewer TE subfamilies and loads than the remaining species. In *T. urticae*, TIR is the most abundant class (5.8%), followed by the LTR class (2.9%), while the remaining two classes (LINE and SINE) are quite scarce (<0.5%). A total of 12.4% of the genome of this species consists of TEs (with 2% simple repeats), and the distribution of the sequence divergence presents two distinct peaks (Figure 2a), which is very different from the other species (Figure 2b–d). The first peak in *T. urticae* is from 0% to 1%, and the second one is from 20% to 24%. LTRs are the major component of the first peak, which implies that there is a recent expansion of LTRs in *T. urticae*. For instance, the *Gypsy-6_TetUrt* has at least eight full-length copies. In contrast, the second peak mainly consists of TIRs, which suggests that most TIRs expanded historically and might have diverged thereafter.

#### 3.2.3. Lepidopterans Have Diversified Transposable Elements Subfamilies and Large Transposable Elements Loads

For all four *Lepidoptera* species (*Bombyx mori, Melitaea cinxia, Papilio xuthus,* and *Spodoptera frugiperda*), there are hundreds of TE subfamilies in their genomes. Among these species, *B. mori* has the most abundant TE subfamilies and the largest TE loads (Figure 1c). More than half (51.26%; Table S1) of its genome is occupied by TEs, which is consistent with a previous report [11]. Compared with the three-remaining species, *B. mori* also has the largest genome (Table 1). In addition, the TE divergence distributions of this species show distinct patterns. In *B. mori*, LTRs, TIRs, and LINEs all fall into the 1–10% range, especially the LTRs, almost all of which are located in the 1–5% range (Figure 2b). These results suggest that most LTRs are active and have expanded recently in *B. mori*. However, a distinct distribution is observed in *S. frugiperda*, the evolutionarily closest species to *B. mori* among the 14 selected species. The distribution resembles a classical normal distribution with a peak near 10% (Figure S2A). A similar distribution is observed in *M. cinxia* (Figure S2B). These results suggest that the expansion of TEs are mostly ancient in both *S. frugiperda* and *M. cinxia.* Notably, *P. xuthus* shows a bimodal distribution of TE divergence: one peak is caused by the recent expansion of TEs (the divergence peak is ~4%), and another peak is caused by the historic expansion of rolling circle (RC) TEs (the divergence peak is ~13%, Figure S2C). Altogether, our results suggest that the TE expansion histories in *Lepidoptera* are diverse.

#### 3.2.4. The Evolutionary History of LTRs in *Lepidoptera*

*Bombyx mori* has the most abundant LTRs among the four *Lepidoptera* species, and the sequence divergence distribution reveals that most LTRs are accumulated due to recent expansion (Figure 2b). However, fewer LTRs are found in the three-remaining species (*M. cinxia, P. xuthus,* and *S. frugiperda*; Figure S2A–C). This finding promotes us to ask when, where and which LTRs were gained or lost in *Lepidoptera*. To further explore the details of the evolutionary history of LTRs, we selected intact LTRs and constructed their phylogenies. We annotated proteins of all LTRs and built phylogenetic trees for all three main LTR subfamilies (*BEL/Pao*, *Copia*, and *Gypsy*).

**Figure 3:**
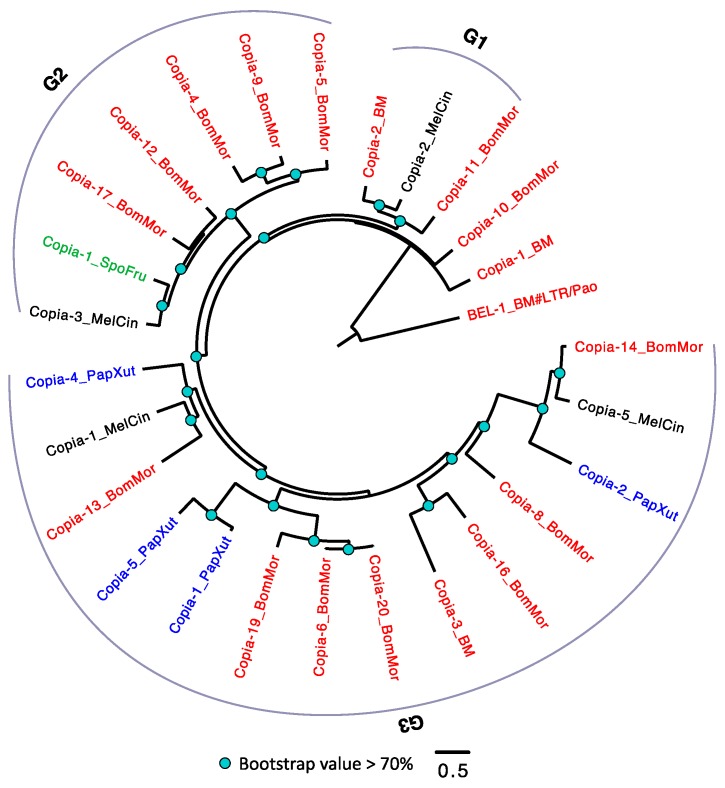
The phylogeny of *Copia* in *Lepidoptera*. The *Copia* were arbitrarily divided into three groups (G1–3). TEs with BM (annotated in Repbase) or BomMor (annotated in this study) suffixes are from *B. mori* (red name); TEs with PapXut, MelCin, and SpoFru suffixes are from *P. xuthus* (blue name), *M. cinxia* (black name), and *S. frugiperda* (green name). Only nodes with bootstrap value not lower than 70% were indicated. *BEL-1_BM* is the outgroup.

Unlike the TEs in *D. melanogaster* that had been well studied and classified into detailed subfamilies [62,63], the family information of LTRs in *Lepidoptera* are still lacking. Therefore, we arbitrarily divided the *Copia* into three groups (G1–3) according to the phylogeny (Figure 3). Among the four species, *S. frugiperda* has the smallest number of *Copia* (only one member in G2), while *B. mori* has the largest number of *Copia*. Within each group, we frequently detected expansion of *Copia* in *B. mori.* For example, we observed a pair of TEs (*Copia-6_BomMor* and *Copia-20_BomMor* in G3) with extremely low amino acid substitution level in *B. mori*, suggesting that these two could be recently duplicated. Although each of the four species has *Copia* in their genomes, none of the three groups has *Copia* detected in all the four species. A parsimonious explanation is that some *Copia* copies might be lost in a species-specific manner, although we cannot exclude the possibility that the phylogeny is solely caused by TE duplications followed by sequence diversifications.

**Figure 4:**
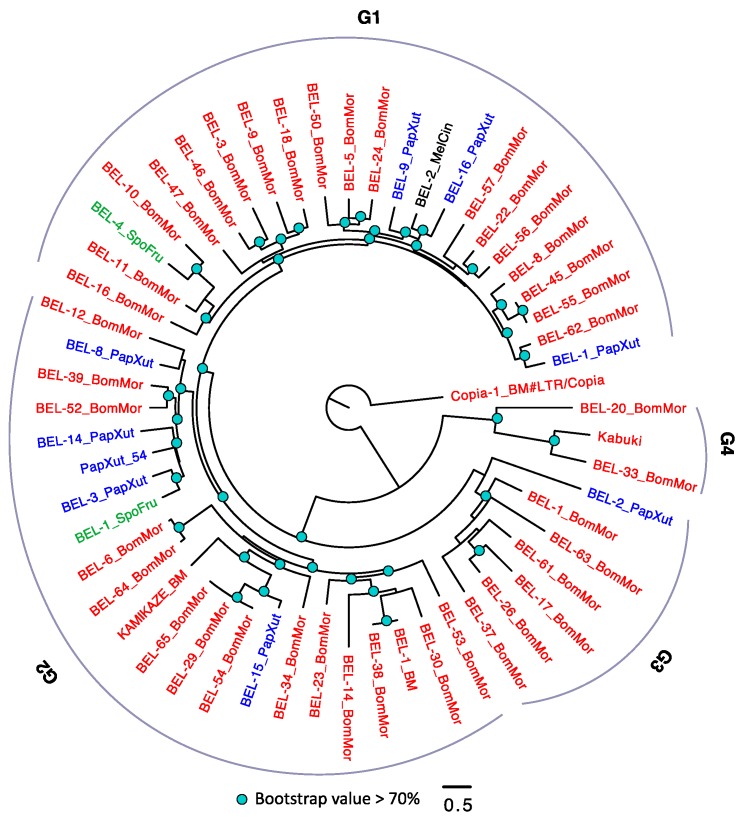
The phylogeny of *BEL/Pao* in *Lepidoptera*. The *BEL/Pao* were arbitrarily divided into four groups (G1–4). TEs with BM (annotated in Repbase) or BomMor (annotated in this study) suffixes are from *B. mori* (red name); TEs with PapXut, MelCin and SpoFru are from *P. xuthus* (blue name), *M. cinxia* (black name) and *S. frugiperda* (green name). *Kabuki* is from *B. mori* and has been annotated in *BmTEdb* [64]. Only nodes with bootstrap value not lower than 70% were indicated. *Copia-1_BM* is the outgroup.

In the *BEL/Pao* subfamily phylogeny, there are four large groups (Figure 4). Similar to *Copia*, *B. mori* also has the largest number of TEs, followed by *P. xuthus*. We frequently observed intraspecific diversifications of *BEL/Pao* (with extremely small amino acid substitution rates) in *B. mori*. It is also possible that the disparity of *BEL/Pao* contents in the four species are caused by the loss of *BEL/Pao* in certain species, although further studies are required to verify this pattern.

**Figure 5:**
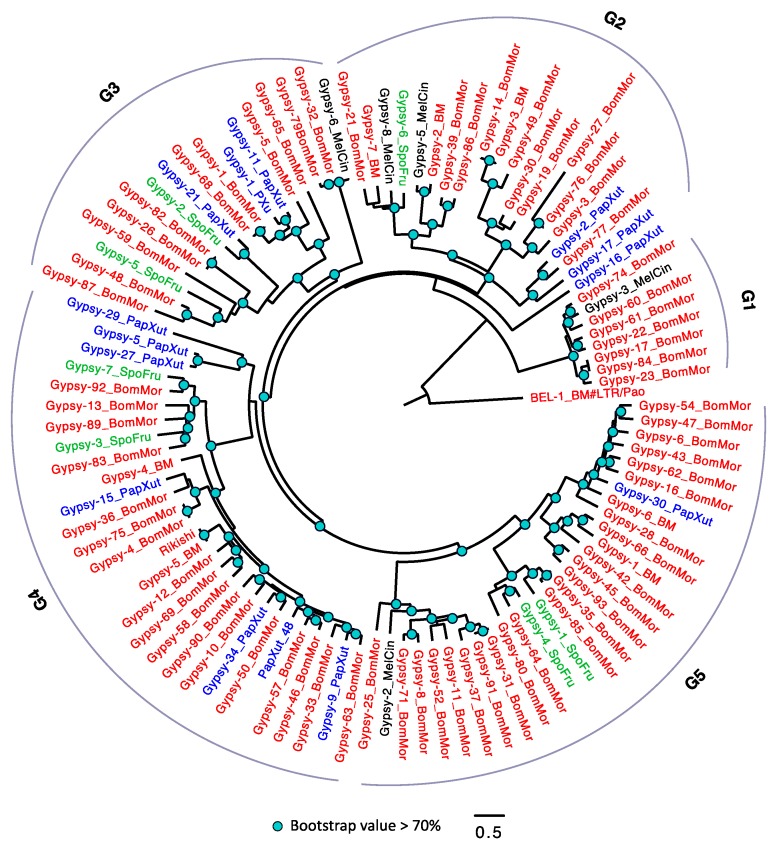
The phylogeny of *Gypsy* in *Lepidoptera*. The *Gypsy* were arbitrarily divided into five groups (G1–5). TEs with BM (annotated in Repbase) or BomMor (annotated in this study) suffixes are from *B. mori* (red name); TEs with PapXut, MelCin and SpoFru are from *P. xuthus* (blue name), *M. cinxia* (black name) and *S. frugiperda* (green name). *Rikishi* is from *B. mori* and has been annotated in *BmTEdb* [64]. Only nodes with bootstrap value not lower than 70% were indicated. *BEL-1_BM* is the outgroup.

The comparison of the TE contents and loads of *Lepidoptera* revealed that *Gypsy* was most abundant among the three LTR families (Figure 1c, Figure 2b and Figure S2A–C). This family might have the most substantial effect during LTR evolution in *Lepidoptera*. The *Gypsy* phylogeny (Figure 5) reveals three major conclusions. First, *B. mori* has the most abundant *Gypsy* TEs. Second, there are extensive diversifications of *Gypsy* in *B. mori* and *P. xuthus*. Third, we observed one horizontal transfer event between *B. mori* and *M. cinxia* (in G3). The amino acid distance between *Gypsy-6_MelCin* and *Gypsy-32_BomMor* is small (0.052). Considering the long divergence time between *B. mori* and *M. cinxia* (the amino acid distance cutoff between these two species is 0.065; Table S3), the small amino acid distance mostly conflicts with the evolutionary history, confirming the HTTs might be *bona fide*.

Altogether, our results suggest that LTRs evolve expansions and contractions, or intraspecific sequence diversification. All these processes combined to form the current LTR patterns in *Lepidoptera.*

#### 3.2.5. Non-LTR Transposable Elements Contribute More to Arthropods with Larger Genomes

Given that TEs exist in and occupy large sections of *Arthropod* genomes, we ask how the genome sizes dynamically change due to TE difference. Interestingly, we found the content of TIR (%) in one arthropod genome is positively correlated (Pearson’s product-moment correlation: 0.899, *p* < 0.0001) with the genome size (Table S4). In addition, the LINE superfamily shows high abundance in *L. migratoria* (Figure 1c), which may have resulted from the recent rapid expansion of this family (Figure 2c). When the divergence distribution is considered, in *L. migratoria* and *A. geniculata*, the peaks are all close to 6% (Figure 2c,d), suggesting that most of the identified TEs have accumulated recently during their evolutionary history. This pattern suggests that most TEs in their genomes were recently generated through rapid TE expansions.

Altogether, our results show that TE loads vary greatly among arthropods and reveal that the gain and loss of non-LTR TEs are much more prevalent in arthropods than previously thought. The very recent study reported similar conclusions [19], while the underlying causes of diversified TE and lineage-specific activity were not mentioned. Here, we analyzed the TE dynamics of species from the same orders (*Hymenoptera* and *Lepidoptera*) or with extremely large genome sizes. The results reveal that rapid extinction, intraspecific diversification, and HTT are the internal driving forces of the diversity of TEs in arthropods.

### 3.3. The Expansion and Contraction of Transposable Elements in Arthropods

RNAi is a critical mechanism to inhibit TEs transposition and hence reduces TE loads in metazoans [65,66]. Besides RNAi, the difference in life history, mating system, GC contents have been suggested to account for the difference in TE loads across species [51]. However, the study of long-term TE evolution in 42 nematode species suggest that genetic drift rather than life history or RNAi mainly determined the evolution of TEs [51]. In addition, the authors tested load changes of four main TE classes and found several “expansion hotspots” in the most dynamic LTR TEs [51].

Given the disparity of TE loads across the 14 arthropod species we studied, here we explore the expansion and contraction of TEs in arthropods. We first used phylogenetic comparative analysis to explain the TE diversity over long-time scales. The phylogeny of the fourteen-selected species was inferred using single-copy orthologous genes and time-calibrated using r8s [41]. We evaluated the phylogenetic signals of traits (TE loads and genome sizes transformed in natural log(Ln)grams) using the ***λ*** model with phytools [43,50]. The ***λ*** is from 0 to 1, and greater values imply stronger phylogenetic signals (trait is highly related to the phylogeny and not random). Most of the kept traits (24/27) have ***λ*** values larger than 0.5 (Table S2), indicating that they are highly associated with the phylogeny. To determine the most appropriate comparative model, we fitted four standard phylogenetic comparative models (BM, OU, EB, and white) for the above traits using Geiger. We used both AICc and the weighted AICc (AW) to assess the fitness of the four models. The results showed that the BM model was most suitable (Table S2). Therefore, TE loads of ancestor nodes were inferred using the maximum likelihood method [67] under the BM model.

**Figure 6:**
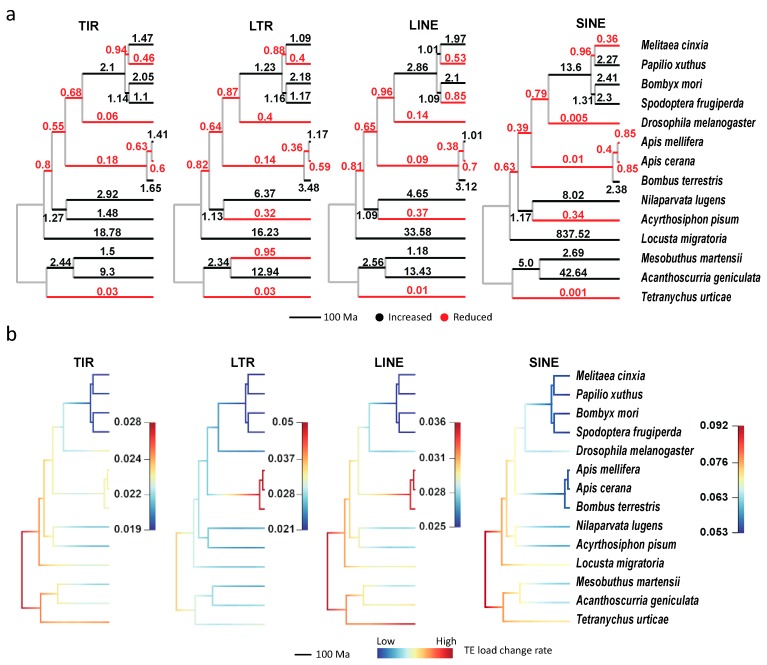
The expansion and contraction of TEs in the 14 arthropod species. (**a**) The ratio of TE load change (the offspring relative to the ancestral node) for each branch. (**b**) The dynamic evolutionary rates of TE loads in the phylogeny. Branch colors are scaled by evolutionary rates of TE loads, and rate increases from the cold color (blue) to warm color (red). TIR, terminal inverted repeat; LTR, long terminal repeat; LINE, long interspersed nuclear element; SINE, short interspersed nuclear element.

We found TE expansion (the black branches) and contraction (the red branches) hotspots in all four main TE families (Figure 6a). Although the expansion hotspots (with large change ratios) are slightly different among the four TE classes, most of them are enriched in *L. migratoria*, *A. geniculata*, *N. lugens* and *B. mori*, which is consistent with their TE divergence distributions (Figure 2b–d). Moreover, the genomes of all four species are larger than those of closely related species. Besides, the TE contraction also broadly exists in all four classes, and the most significant species are *T. urticae*, *D. melanogaster,* and the three bees.

The above results suggest that the changes of TE loads were highly variable during arthropod evolution. Therefore, we applied BAMM to analyze the dynamical change of evolutionary rates for TE loads under the phylogeny. In Figure 6b (so-called phylorate plots), although no shift of TE loads evolutionary rates is in all the four classes, the evolutionary rates are broadly variable among both TE classes and clades. Among the four classes, SINE has the highest rates, while TIR has the lowest ones, which helps to explain the largest TE loads variants of SINE (Figure 1c) in arthropods. The rates in *Lepidoptera* clades are broadly lower than the remaining ones, consisting with their low TE loads changes (Figure 6a) relative to ancestor nodes. Besides, three branches (the root branch, *T. urticae,* and *Hymenoptera* clades) have higher rates than the other branches in TIR, LTR, and LINE classes (the root branch and *T. urticae* clade are also higher in SINE). In addition, evolutionary rate dynamics of three representative subfamilies (Figure S3; *Jockey*, *Mariner*, and *Gypsy*) were also evaluated, and the results also supported that evolutionary rates were high in the *Hymenoptera* clade (shift events were identified in *Jockey* and *Gypsy*). These results reveal that the TE loads had rapidly changed at the early radiation of insects and arachnids, and the TE loads change rates could be branch-specific instead of underlying constraints.

### 3.4. Transposable Element Loads are Significantly Correlated with Genome Sizes

Transposable element contents have been reported positively correlated with the genome sizes in eukaryotes [20,68]. TE contents and the number of subfamilies were found to be correlated with host genome sizes in arthropods [19], indicating that the genomes with larger sizes also have greater TE contents or more TE subfamilies. However, TE contents and the subfamily numbers might be a little more variable than TE loads due to the influences of the large variation of host genome sizes in arthropods. Here, we evaluated correlations between TE loads and TE contents and genome sizes. The results showed that both of these two are positively correlated with genomes sizes and the TE loads (Pearson’s product-moment correlation: 0.92; *p* = 2.76 × 10^−6^; Figure S4A) have a greater correlation coefficient than TE contents (0.71; *p* = 4.92 × 10^−3^; Figure S4B).

The quantitative traits in species from a branching phylogeny are not statistically non-independent due to common ancestry, and the PICs should be conducted [69]. We evaluated the correlations between loads of TEs from multiple subfamilies and host genome sizes. As shown in Table 2, our results show that many TE subfamilies are positively correlated with host genome sizes. Especially, the most abundant *Mariner* (TcMar) subfamily is significantly correlated with the corrected *p* < 0.1. In addition, we also evaluated the correlation between the total TEs of four main families and genome sizes. All the four classes are positively correlated with host genome sizes, for which the corrected *p* < 0.1 (Table S5). After combining all TEs in each species, we observed that total TE loads were also significantly correlated with genome sizes (Pearson’s product-moment correlation: 0.83; *p* = 4.74 × 10^−4^; Table S5). This result is consistent with the PIC analysis in a recent study [19]. Unlike the TE contents used in the previous study, we used TE loads which might reflect TE abundance in host genomes a little better. Although only 14 species used in this study, the TE loads showed a much stronger correlation than TE contents. Our results reveal that TE expansion is one of the most critical forces driving changes in the sizes of host genomes, which is also consistent with previous reports in flies [22], birds and mammals [21].

### 3.5. Horizontal Transposable Element Transfer in Arthropods

TEs can be transferred by vertical inheritance or horizontal transfer. *P-element* is the first reported and the most famous HTT in *Drosophila* [70] and is the genetic basis of P-M hybrid dysgenesis in *D. melanogaster* [71]. HTT is associated with many phenotypic changes in plants [72]. A recent study identified thousands of HTT events in Insecta [73], and these transferred TEs might be important in driving genome evolution [74]. Here, we identified millions of TE copies from thousands of subfamilies in fourteen arthropods. We asked whether HTTs had been involved in TE evolution, especially in species with large genomes. To solve this problem, we proposed a strategy with which to identify HTTs using genome-wide amino acid distances. Amino acid distance has been widely used in phylogenetic studies, and a smaller distance implies a higher sequence similarity. We expected the amino acid substitution rate of TE (evolving mostly neutrally or being counter selected, thus being permissive to *dN* substitutions) to be higher than orthologous genes (more conserved with a higher rate of negative selection). Therefore, the vertically inherited TEs will have larger amino acid distances than orthologous genes. On the contrary, TEs with lower amino acid distances could be HTTs.

Considering the variable quality of gene annotations in the selected species, we used the BUSCO tool with the built-in Insecta core genes library and annotated genes in all selected species. Using the single-copy homologous genes, we inferred the genome-wide amino acid distance cutoffs (Table S3; see details in Materials and Methods) for each pair of fourteen species. Annotated TE proteins were aligned between each pair of species. The amino acid distances of aligned TE pairs were calculated and compared with the genome-wide cutoff, which was defined as the 100th minimum amino acid distance between two species. According to the recent study in 76 arthropods, the number of genes that present in more than 75% Metazoans are all larger than 5000 [60], which implies that the number of orthologous genes between most arthropods might be larger than 5000. In this study, we set the genome-wide cutoff as the 100th minimum amino acid distance between two species, which equals to at most 2% of the total orthologous genes.

**Figure 7:**
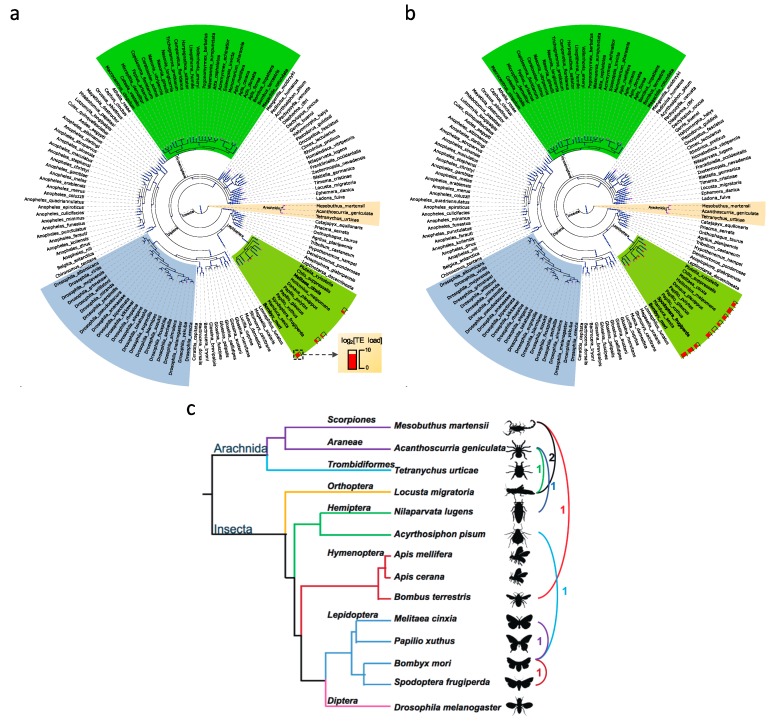
The HTTs in arthropods. (**a**) The HTT of *Gypsy* between *M. cinxia* and *B. mori*. (**b**) The HTT of *Mariner* between *B. mori* and *S. frugiperda*. The species names of hosts of HTT are in bold. (**c**) Eight HTTs in the fourteen arthropods.

We obtained eight candidate HTTs among nine species (Figure 7a,b and Figure S5; Table S6), and their protein alignments are in Supplementary File S2. All of them are best reciprocal hits between corresponding species. Two of them are from the *Gypsy* subfamily. Six of them are TIRs from the *hAT-Tip100* (n = 1) and *Mariner* (n = 5) subfamilies. The Class II (TIR) TEs are the most frequent HTTs, consisting with previous reports in arthropods [73,75]. The Class II TEs tend to be shorter than Class I TEs, besides their transpositions have weak host dependence [76]. These might help to explain why the Class II (especially the *Mariner* subfamily) have the most abundant HTTs.

In Figure S6A–C, two HTT examples are depicted. These two HTTs all have amino acid distances (the blue line) lower than the genome-wide cutoffs (the gray line). The horizontal transfer event between *Gypsy-6_MelCin* and *Gypsy-32_BomMor* is confirmed because the amino acid distance between them is 0.054, which is lower than the cutoff (0.065) between *B. mori* and *M. cinxia*, which is consistent with the phylogenetic analysis of *Gypsy* in *Lepidoptera* (Figure 5). Besides, these two TEs only appear in four of 140 arthropods (Figure 7a), which implies that the high sequence similarity between them are resulted by horizontal transfer instead of vertical inheritance. Another example is the *Mariner* HTT between *B. mori* and *S. frugiperda* (Figure 7b). The amino distance between TEs is 0.034 which is lower than the genome-wide cutoff 0.047 (Figure S6C; Table S3).

The exact number of HTTs between these species might be underestimated (i) because several TEs were not annotated due to the methodological limitations and low-quality genome assemblies and (ii) because of the strict cutoffs used in HTT identification. Besides, six HTTs were identified in five species (*A. pisum, A. geniculata*, *L. migratoria*, *M. martensii,* and *N. lugens*) with genomes larger than 500 Mb (Figure 7c). Considering the large genomes and the TE loads in these species and other arthropods, our and previous results [73,75] both support that HTTs played important roles during their genome expansions and might be another important driving force of genome evolution.

### 3.6. The ArTEdb

Database technology has been widely used in biological data sharing, especially in the field of TE research (recently reviewed [77]). Several databases provide TE annotations or repeat-masking results [23,24]. Here, to make new TE references and annotations easy to use and contribute to TE studies in arthropods, we set up the ArTEdb. It consists of four main categories: (1) TE landscapes in 14 arthropods, (2) TE references, annotations and downloading of premasked genomes, (3) TE querying based on keywords and sequence similarity, and (4) an online repeat-masking service.

We summarized TE annotations and generated an overview of TE contents for each species. The TE contents were organized by both subfamilies and sequence divergence between each TE copy and TE reference. We provide two methods for querying TEs. People can look up TEs by name, subfamily, and class. Alternatively, one can also use a sequence to search the TE database directly with BLAST. The genome browser helps visualize genomic features more intuitively. We integrated the JBrowse genomic feature visualization tool into the ArTEdb. Using this tool, people can focus on genes or other genomic loci rather than masking repeats by themselves. In addition, we provide an online repeat-masking service, which is very useful for people who want to scan TEs from just a small number of sequences quickly. Finally, all TE annotations and premasked genomes can be downloaded from the ArTEdb directly. We hope that the ArTEdb will benefit future TE studies in arthropods.

## 4. Discussion

In this study, we chose 14 representative arthropods spanning eight orders to study the coevolution between TEs and host genomes. Using our customized TE annotation pipeline, we both generated high-quality TE references and estimated the TE profiles in these species.

### 4.1. A New Database of High-Quality Transposable Element References for Arthropods

Characterizing TEs in an unbiased approach is an important task for the non-model organisms. Although Repbase is extensively used for TE identification and annotation [23], the TE reference sequences might not be complete for many non-model organisms. Therefore, many TE annotation tools have recently been developed for unbiased TE characterization [26,27,28,31,77]. In this study, we built an unbiased TE annotation pipeline (Figure 1a) by combining different TE identification and classification tools. We also present our reference sequences in the ArTEdb database, which provides useful resources for TE annotations in other arthropod genomes.

A very recent study [19] have extensively characterized TEs in the arthropod genomes with the RepeatModeler pipeline. RepeatModeler aims to identify sequences with high-copy numbers in genomes, which is suitable for identifying most types of TEs. However, RepeatModeler does not take into account the structural characteristics of TEs, and sometimes it does not perform as well as the family-specific tools. For example, the novel TE *Gypsy-1_DroMel* was identified by both RepeatModeler and two LTR-specific tools (LTR_Finder and LTRharvest). The sequence alignment indicates that RepeatModeler only identified a partial sequence of *Gypsy-1_DroMel,* while the combined approach we used (combining RepeatModeler, LTR_Finder, and LTRharvest) successfully identified the full-length *Gypsy-1_DroMel* (Figure S1). Besides TE identification, our ArTEdb database also significantly improved the annotation of TE family information. As RepeatModeler only uses sequence similarity to known TEs in TE classification, most TEs identified by RepeatModeler remain unclassified [19]. In this study, we also used PASTEClassifier [32] and TEclass [33] to annotate the identified TEs. PASTEClassifier takes full use of known TEs (Repbase) and domain annotations (Pfam and Gypsydb), and TEclass utilizes the supporting vector machine based on oligomer frequencies of repeats. These comprehensive classification methods make almost all the TEs be classified at least in class-level. Therefore, our study provides TE profiles with better annotation information.

### 4.2. Why Does the Transposable Element Loads Can Be So Different Across Arthropod Species?

Previous studies show that TEs gain and loss are important for genome sizes variation in vertebrates [21] and *Drosophila* [22]. Both a very recent study [19] and our results detected frequent gains and losses of TEs in the arthropod genomes. Moreover, our results suggest that multiple evolutionary forces can cause TE profiles to be very different even between closely related species, as shown in the four *Lepidoptera* species we analyzed (Figure 3,Figure 4,Figure 5). Unlike the protein-coding genes which are in general under selective constraints, the sequences of TEs are usually less constrained and evolve rapidly [78]. Therefore, besides the copy number variation caused by TE expansion and contraction, sequence diversification between homologous TE can also lead to TE diversification in the arthropods. Moreover, HTT is not uncommon between arthropods as shown in our results and previous studies [73,75]. Therefore, our results suggest that the TE profiles in the arthropod genomes are shaped by expansion and contraction of TEs, TE sequence diversification, and HTTs.

Then why can the TE loads be so different across arthropod species? According to the classic population genetics framework of TE biology, the content of TEs in a species is shaped by its rapid replication and the selective constraints because TEs are in general deleterious and selected against in most species. Since the selective strength of TEs in a species is determined by the effective population size (*Ne*) of that species [79], it is possible that the difference in *Ne* across arthropod species might be important for the variation in TE loads. In addition, DNA methylation [80] and RNA interference pathway [81,82] also suppress TE activities in arthropods, and the suppressive effects of both mechanisms might vary in arthropods. Therefore, the vast difference in TE loads among arthropods can be caused by the difference in natural selection or in the epigenetic regulatory mechanisms, although at this moment we cannot exclude the possibility that the TE difference in the studied arthropod species is mainly shaped by genetic drift as previously shown in nematode [51].

### 4.3. The Contribution of HTTs to the Transposable Element Repertoire in Arthropod Genomes

Previous studies have reported numerous HTTs in arthropods [70,73,75] and plants [83] (details are reviewed in Ref. [72,84]). In arthropods, most HTTs occurred for the *Mariner* (TcMar) subfamily [73,75], which are generally shorter (1–2 Kb) than LTRs (mostly longer than 5Kb) and might be easily transferred by vectors [85]. Accordingly, among the eight HTTs identified in this study, five of them are caused by the *Mariner* subfamily. Notably, the species with larger genomes have greater TE loads and more HTT events, suggesting that HTT may contribute to the TE expansions. In eukaryotes, TEs are repressed either by suppressing TE transcription or by piRNA-mediated cleavage of TE transcripts [86,87]. As the host organisms take time to develop piRNAs to repress a newly horizontally transferred TE, that TE might replicate rapidly and contribute significantly to the TE repertoire until abundant piRNAs are developed to repress that TE [82].

TEs recently horizontally transferred between two species will have higher sequences similarity than the protein-coding genes, which cause *dS* values to be smaller for the TEs than that for the protein-coding genes between the two species [73,88]. In this study, we used the amino acid distance instead of the *dS* to identify HTTs in arthropods, because the synonymous substitutions in the protein-coding genes between the studied species are usually saturated (*dS* values usually > 1). Since the number of orthologous genes between every two arthropods might be larger than 5000 [60], and our BUSCO analyses might have captured the most conserved ones, therefore, we set the genome-wide cutoff as the 100th minimum amino acid distance between two species, which represents the top ~2% of the total orthologous genes (Table S3). Thus, the HTTs we identified in this study do not necessarily recently occur, but might have occurred anciently.

### 4.4. Adaptive Transposable Element Insertions in Arthropods

In recent decades, numerous studies have demonstrated that TEs can benefit their hosts in multiple ways. For instance, TEs could be domesticated as promotors [89,90,91] or enhancers [92,93,94] to regulate gene expression. Moreover, a few TEs could be co-opted into novel protein-coding genes in the host genomes [95,96,97,98,99,100]. These findings suggest that TEs are important for providing raw materials of the regulatory elements and proteomes for the hosts. In addition, many studies have shown that TE insertions might increase the fitness of hosts. For example, a *P* element insertion in the promoter of *Hsp70A* significantly increases the fecundity of heat-shocked flies [91], and a *Doc1420* insertion in *CHKov1* significantly increases the pesticide resistance of hosts by disrupting the original gene structure [101]. The carbonaria form of the peppered moth (*Biston betularia*) was reported to be caused by a TE insertion [102] that upregulates a cortex transcript involved in early wing disc development. All these studies indicate that the beneficial effects of TEs are pervasive in eukaryotes. Although millions of TEs had been annotated in our study, the beneficial TE insertions remain unknown. Thus, further studies are required to identify the role of TEs in the adaptation of arthropods.

## Figures and Tables

**Table 1:** Genome sizes and the number of transposable elements references of fourteen arthropods.

Class	Order	Species	Genome Sizes ^†^	TE Reference Sequences		
				RB/RM/LTR	RTE/DTE/UnC	Total
***Insecta***	***Diptera***	***Drosophila melanogaster***	144 Mb	147/79/8	159/47/28	234
	*Lepidoptera*	*Spodoptera frugiperda*	358 Mb	1/475/12	263/190/35	488
		*Bombyx mori* ^‡^	460 Mb	92/546/183	552/232/37	821
		*Papilio xuthus*	244 Mb	41/319/60	248/151/21	420
		*Melitaea cinxia*	390 Mb	0/763/17	433/299/48	780
	*Hymenoptera*	*Bombus terrestris*	249 Mb	6/520/12	241/267/30	538
		*Apis cerana*	228 Mb	0/86/1	28/49/10	87
		*Apis mellifera*	250 Mb	6/136/1	35/98/10	143
	*Hemiptera*	*Acyrthosiphon pisum*	542 Mb	331/752/75	326/796/36	1158
		*Nilaparvata lugens*	1.14 Gb	0/1,136/230	872/419/75	1366
	*Orthoptera*	*Locusta migratoria*	5.6 Gb	1028/1182/56	1144/1018/104	2266
*Arachnida*	*Trombidiformes*	*Tetranychus urticae*	90 Mb	10/122/73	105/86/14	205
	*Araneae*	*Acanthoscurria geniculata*	7.2 Gb	0/1857/167	967/982/75	2024
	*Scorpiones*	*Mesobuthus martensii*	925 Mb	39/1260/101	476/843/81	1400

^†^ Sizes of current versions of genomes used in this study. ^‡^ Recently assembled genome based on PacBio single-molecule sequencing datasets. RB, RM, and LTR symbolize Repbase, RepeatModeler, and LTR *de novo*, respectively. RTE, DTE, and UnC symbolize retrotransposable elements, TIRs, and unclassified TEs, respectively.

**Table 2:** TE loads are significantly correlated with genome sizes.

TE Subfamilies	Correlation Coefficient	*P*	Corrected *P*
LINE/L2	0.868	1.20 × 10^−4^	3.01 × 10^−3^
SINE/Unclassified	0.784	1.51 × 10^−3^	3.18 × 10^−2^
LINE/Unclassified	0.760	2.55 × 10^−3^	4.85 × 10^−2^
DNA/Unclassified	0.756	2.79 × 10^−3^	5.02 × 10^−2^
DNA/TcMar	0.728	4.75 × 10^−3^	8.07 × 10^−2^
LTR/Copia	0.724	5.14 × 10^−3^	8.22 × 10^−2^
DNA/hAT	0.654	1.53 × 10^−2^	2.13 × 10^−1^
LINE/CR1	0.644	1.75 × 10^−2^	2.13 × 10^−1^
LINE/RTE	0.652	1.57 × 10^−2^	2.13 × 10^−1^
LTR/Unclassified	0.654	1.52 × 10^−2^	2.13 × 10^−1^
LTR/Pao	0.644	1.76 × 10^−2^	2.13 × 10^−1^
DNA/Ginger	0.559	4.72 × 10^−2^	4.25 × 10^−1^
LINE/I	0.511	7.45 × 10^−2^	5.96 × 10^−1^
DNA/CMC	0.489	8.97 × 10^−2^	6.28 × 10^−1^
DNA/Academ	0.419	1.54 × 10^−1^	9.23 × 10^−1^
LINE/Dong-R4	0.403	1.73 × 10^−1^	9.23 × 10^−1^
DNA/Harbinger	0.358	2.30 × 10^−1^	9.23 × 10^−1^
DNA/PiggyBac	0.371	2.12 × 10^−1^	9.23 × 10^−1^
LINE/Jockey	0.394	1.83 × 10^−1^	9.23 × 10^−1^
LINE/R1	0.351	2.40 × 10^−1^	9.23 × 10^−1^
LTR/Gypsy	0.382	1.98 × 10^−1^	9.23 × 10^−1^

Unclassified: subfamily information is unavailable.

## Supplementary Materials

The following are available online at https://www.mdpi.com/2073-4425/10/5/338/s1, Figure S1. The comparison of one TE identified by LTR-specific tools and RepeatModeler. Figure S2. Sequence divergence distribution of TEs in three *Lepidoptera* species. Figure S3. Rate shifts of TE loads in the phylogeny. Figure S4. The correlation between TEs and host genomes sizes. Figure S5. The copy numbers of six HTTs in 140 arthropods. Figure S6. Two HTTs in arthropods. Table S1. Basic information about the fourteen species. Table S2. The weighted small sample size corrected AIC (AW) and *λ* of the traits. Table S3. Cutoffs of amino acid distances. Table S4. The content of TIR in the 14 arthropod genomes. Table S5. TE loads are significantly correlated with genome sizes. Table S6. Eight horizontally transferred TEs. File S1. The phylogenetic tree of fourteen species. File S2. Protein alignment of eight HTTs.
