# Supplementary material for: Diversification of Transposable Elements in Arthropods and Its Impact on Genome Evolution
Source: Genes (Basel). 2019 May 6;10(5):338. doi: 10.3390/genes10050338 (PMC6562904; doi:10.3390/genes10050338)
Supplement: Supplementary file 1 [file genes-10-00338-s001.zip › genes-456725-supplementary/File S2.docx]

**Protein sequences alignment of eight HTTs.**

**01. Gypsy-60_BomMor#LTR/Gypsy *vs* Gypsy-25_AcyPis#LTR/Gypsy**

Expect Identities Positives Gaps

0 572/743 (76%) 668/743 (89%) 2/743 (0%)

Gypsy-60_BomMor 2 MDRYCSKRPYRCTIEDKKEIETQIAKLLDNKLIEESYSPFAAPITLAFKKEENKRSRLCIDFREL 66

+D+YCSKRPYRC++EDK EIE QI+KLL+ KLIE SYSPFAAP+TLAFKKE+ +RSRLCIDFR+L

Gypsy-25_AcyPis 2 VDKYCSKRPYRCSVEDKNEIEEQISKLLEKKLIEVSYSPFAAPVTLAFKKEDKRRSRLCIDFRDL 66

Gypsy-60_BomMor 67 NKIVVPQAQPFPLIEDLILKTRNCNFFSTLDINSAFWSIPLRIEDKNKTGFVTQDGHYQWTCLPF 131

NKIVVPQAQPFPLI+DL++KTRNCN+FSTLDINSAFWSIPLR+ED+ KT FVTQ+GH+QWTCLPF

Gypsy-25_AcyPis 67 NKIVVPQAQPFPLIDDLVIKTRNCNYFSTLDINSAFWSIPLRMEDRKKTAFVTQEGHFQWTCLPF 131

Gypsy-60_BomMor 132 GLKTSPAIFQRILHNILRKYKLTDFTVNFIDDILIFSKTFSDHINHLTQLFEAIKTEGFRLKFTK 196

GLKTSPAIFQR+L ILRKYKLT+F VN+IDDILIFSK F DHI+HLTQL++AI+TEGFRLKFTK

Gypsy-25_AcyPis 132 GLKTSPAIFQRVLSTILRKYKLTEFAVNYIDDILIFSKNFYDHISHLTQLLKAIQTEGFRLKFTK 196

Gypsy-60_BomMor 197 CTFASDSVKYLGHIIQKNCIKPVNDNLISIKDFPVPRTQKNIRQFLGKINFYHGFIPNRAKILDP 261

CTFA+DSVKYLGHIIQ N I+PV DNL+SI +FPVP+TQKNIRQ+LGKINFYH +IPN A ILDP

Gypsy-25_AcyPis 197 CTFATDSVKYLGHIIQNNSIRPVKDNLVSINNFPVPKTQKNIRQLLGKINFYHEYIPNSAIILDP 261

Gypsy-60_BomMor 262 LHNLLKKNQKFIWSDKCQEAFETVKTLLCSQPILEIFDPYLPINIYTDASLEGIGAILKQRQTDG 326

LHNLL+KNQ+F+WS +CQ+AFE +K LLCSQP+LEIFD LPINIYTDASLEG+GAILKQ Q +G

Gypsy-25_AcyPis 262 LHNLLRKNQRFVWSIECQKAFENIKKLLCSQPVLEIFDKDLPINIYTDASLEGVGAILKQTQLNG 326

Gypsy-60_BomMor 327 TEKPVAYFSKKLNESLRKKKAIYLECLAIKEAVRYWQYWLIGKSFTVYSDHKPLEKMNLKSRTDE 391

EKPVAYFSKKLN + +KKKAIYLECLAIKEAVRYWQYWLIGKSFTVYSDHKPLE MN+KSRTDE

Gypsy-25_AcyPis 327 KEKPVAYFSKKLNAAQKKKKAIYLECLAIKEAVRYWQYWLIGKSFTVYSDHKPLENMNIKSRTDE 391

Gypsy-60_BomMor 392 ELGDLTYYLSQYEFQIKYAPGKKNLEADCLSRNPVLESDKNTDELLKVVNLVKLQDILIDQNKNE 456

ELGD+TYYLSQY+FQIKYAPGK NLEADCLSRNPVLE+++N +E LK+VNL+KL DIL DQ NE

Gypsy-25_AcyPis 392 ELGDFTYYLSQYDFQIKYAPGKDNLEADCLSRNPVLETNENKEEQLKIVNLIKLDDILTDQKNNE 456

Gypsy-60_BomMor 457 DIQRNKNKLIEKNKVYLRKVRKREKIIISEEFSIKLLKEVHKNFCHIGIGQMQKTISPYYTAKNL 521

++++ KNKLI+KN VY++K +KR+KI++SEEFSIKL+K +H+N+CHIGI QMQK I YTAKNL

Gypsy-25_AcyPis 457 EVRKKKNKLIKKNNVYFKKCKKRKKIMLSEEFSIKLIKNIHENLCHIGIKQMQKKIGTVYTAKNL 521

Gypsy-60_BomMor 522 MNNIKKVSRSCETCIKNKSRSHDKFGFMSHLGPAKKPFEIISIDTIGGFGSSRSSKKYLHLLVDH 586

NI + ++C CIKNKSR +DKFG+MSHLGPA+KPFEI+SIDTIGGFG S S KKY+HLLVDH

Gypsy-25_AcyPis 522 TKNIIQFCKNCGICIKNKSRGNDKFGLMSHLGPARKPFEIVSIDTIGGFGGSNSEKKYFHLLVDH 586

Gypsy-60_BomMor 587 FTRYAFILTSKTQNANDFIKLITNITETEDIGILLTDQYPGINSKEFKEFLKDKQITLVFTAVNA 651

FTRYAFILTSKTQ+ANDF+KLI NI +T++IGILLTDQYPGINSKEFK FL +K I +VFTA+N

Gypsy-25_AcyPis 587 FTRYAFILTSKTQSANDFVKLINNIVDTDEIGILLTDQYPGINSKEFKRFLSEKSIQMVFTAINT 651

Gypsy-60_BomMor 652 PFSNGLNERLNQTLINKIRCTINENKKK-AWTTIAKECANKYNDTNHSVTGYPPSYLLYGTDVTV 715

PFSNG+NERLNQTL+NKIRC INE K K AWTT+A+EC N+YNDT+HSVTG+ P YLLYGTDV++

Gypsy-25_AcyPis 652 PFSNGINERLNQTLVNKIRCKINEKKNKLAWTTVAQECVNRYNDTDHSVTGFAPRYLLYGTDVSI 716

Gypsy-60_BomMor 716 VPNELKQGKSNN-DWINDRKIALENTIKSH 744

+P+ELK+GK DWI DRKIA +NTIKSH

Gypsy-25_AcyPis 717 LPHELKEGKKTEMDWICDRKIAYKNTIKSH 746

**02. Gypsy-6_MelCin#LTR/Gypsy *vs* Gypsy-32_BomMor#LTR/Gypsy**

Expect Identities Positives Gaps

2e-56 1011/1065 (94%) 1043/1065 (97%) 15/1065 (1%)

Gypsy-6_MelCin 2 NKKSNMDKYLRPERFDIDPSDSSSTRAWIHWRKTFESFISVQKPTAPETDAQSVPSPEKWDDIKF 66

NKK MD+YLRPERFDIDPSDSSSTRAWIHWRKTFE+FI VQKPTAPETDAQSV PEKWD IKF

Gypsy-32_BomMor 2 NKK--MDRYLRPERFDIDPSDSSSTRAWIHWRKTFENFIFVQKPTAPETDAQSVRPPEKWDSIKF 64

Gypsy-6_MelCin 67 QLLVNHISPNIYTYISEATNYEEAITILQKLYIKPKNLIFARHMLATRKQRPEESIDTYLQALKL 131

QLLVNHISPNIYTYISEATNYEEAITILQKLY+KPKNLIFARHMLATRKQRPEESIDTYLQALKL

Gypsy-32_BomMor 65 QLLVNHISPNIYTYISEATNYEEAITILQKLYVKPKNLIFARHMLATRKQRPEESIDTYLQALKL 129

Gypsy-6_MelCin 132 LSKDCDFAAVDAETNKNDNIRDAFIAGISSHKIRQRLLENLTLTLDQAYNQALSLETAEINSQSF 196

LSKDCDF AVDAETNKND++RDAFI+GISSHKIRQRLLENLTLTLDQAYNQALSLETAEINSQ+F

Gypsy-32_BomMor 130 LSKDCDFVAVDAETNKNDSVRDAFISGISSHKIRQRLLENLTLTLDQAYNQALSLETAEINSQNF 194

Gypsy-6_MelCin 197 NTVSLNAVAAKEPTLISPKTQTVHDETCSSVNNPRRRKCFFCGGQIHPRKNCPAFEKTCQLCNKK 261

NTVSLNAVA KEPTL+S K+QT+H+ETCSSVNNPRR+KCFFCGGQIHPRKNCPAFEKTCQLCNKK

Gypsy-32_BomMor 195 NTVSLNAVAPKEPTLVSSKSQTLHNETCSSVNNPRRQKCFFCGGQIHPRKNCPAFEKTCQLCNKK 259

Gypsy-6_MelCin 262 GHFATVCRSSSKPTNSVVVGTEDISACITAASPSSLRKATVPAYIRGVRAEALLDTGSSISFIND 326

GHFATVCRSSSK TNSVVVGTED+SACITAASPSSLRKATVPAYIRG+RAEALLDTGSSISFIND

Gypsy-32_BomMor 260 GHFATVCRSSSKSTNSVVVGTEDLSACITAASPSSLRKATVPAYIRGIRAEALLDTGSSISFIND 324

Gypsy-6_MelCin 327 SFARLCGLKRKSCKQTISMASLNHTSQVEGQTWQTLKIGNHKYDNVNLLIVKNLCADIIIGHDIL 391

+FARLCGLKRKSC QTISMASLN+TSQVEGQTWQTLKIGNH+YDNVNLLIVKNLCADIIIGHD+L

Gypsy-32_BomMor 325 NFARLCGLKRKSCNQTISMASLNYTSQVEGQTWQTLKIGNHRYDNVNLLIVKNLCADIIIGHDVL 389

Gypsy-6_MelCin 392 EEHSSLEFSFGGPKHPLQVYNVTESSEHPFQVCNVAEASVPAVSLFANVPPNCKPIAIKSRRHSK 456

EEHSSLEFSFGGPKHPLQ VCNVAEASVPAV LFANV PNCKPIAIKSRRH+K

Gypsy-32_BomMor 390 EEHSSLEFSFGGPKHPLQ-------------VCNVAEASVPAVPLFANVSPNCKPIAIKSRRHNK 441

Gypsy-6_MelCin 457 EDSEFIKEEIRNLIAEGVIEESKSPWRAQVLITKSETHRKRLVIDYSQTINRYTELDAYPLPNIE 521

EDSEFIKEEIRNLIAEGVIEESKSPWRAQVLITKSETH+KRLVIDYSQTINRYTELDAYPLPNIE

Gypsy-32_BomMor 442 EDSEFIKEEIRNLIAEGVIEESKSPWRAQVLITKSETHKKRLVIDYSQTINRYTELDAYPLPNIE 506

Gypsy-6_MelCin 522 DLVSKVAKDTFFSLIDLKSAYHQVPILPEERKFTAFEALGNLYQFRRIPFGVTNGVSSFQRTIDW 586

DLVSKVAK+TFFSLIDLK+AYHQVPILPEERKFTAFEALGNLYQFRRIPFGVTNGVSSFQRTIDW

Gypsy-32_BomMor 507 DLVSKVAKNTFFSLIDLKNAYHQVPILPEERKFTAFEALGNLYQFRRIPFGVTNGVSSFQRTIDW 571

Gypsy-6_MelCin 587 IIRKEKLKKTYAYLDDITISGRTLEEHDHNLESFINVAKKYGLTLCIQKCKFSQESINILGYNIQ 651

IIRKEKL+ TYAYLDDITISGRTLEEHDHNLESF+N AKKYGLTL IQKCKFSQ+SINILGYNIQ

Gypsy-32_BomMor 572 IIRKEKLQNTYAYLDDITISGRTLEEHDHNLESFMNAAKKYGLTLSIQKCKFSQKSINILGYNIQ 636

Gypsy-6_MelCin 652 NHIIKPDSERLKPLINLPPPSDLPTLRRTLGMFAHYSKWIPNFSERIHSLANTTTFPLTSEEIKC 716

NHIIKPD+ERLKPLINLPPPSDLPTLRRTLGMFAHYSKWIP FSERIHSLANTT FPLTSE+IKC

Gypsy-32_BomMor 637 NHIIKPDNERLKPLINLPPPSDLPTLRRTLGMFAHYSKWIPKFSERIHSLANTTIFPLTSEQIKC 701

Gypsy-6_MelCin 717 FEGLKNDIAKSSVHAIDENIPFTVETDASDHSIAAVLTQNSRPVAFFSRTLNSSEQKHSAIEKEA 781

FE LKNDIAKSS+HAIDENIPFTVETDASDHSIAAVLTQNSRPVAFFSRTLNSSEQ HSAIEKEA

Gypsy-32_BomMor 702 FESLKNDIAKSSIHAIDENIPFTVETDASDHSIAAVLTQNSRPVAFFSRTLNSSEQNHSAIEKEA 766

Gypsy-6_MelCin 782 YAIVESLKKWRHFLIGRHFKLVTDQRSVSFMFNMKHSSKIKNEKIQRWRLELAVFKYDIIYRPGK 846

YAIVESLKKWRHFLIGRHFKLVTDQRSVSFMFNMKHSSKIKNEKIQRWRLELA FKYDIIYRPGK

Gypsy-32_BomMor 767 YAIVESLKKWRHFLIGRHFKLVTDQRSVSFMFNMKHSSKIKNEKIQRWRLELAAFKYDIIYRPGK 831

Gypsy-6_MelCin 847 ENYAADALSRVCATVETRTTKLFSLHEALCHPGVTRMFHWVRSKNLPYSIEEVRTMTKSCRICSE 911

ENYAADALSRVCATVETRT KLFSLHEALCHPGVTRMFHWVRSKNLPYSIEEVRTMTKSCR CSE

Gypsy-32_BomMor 832 ENYAADALSRVCATVETRTAKLFSLHEALCHPGVTRMFHWVRSKNLPYSIEEVRTMTKSCRTCSE 896

Gypsy-6_MelCin 912 VKPRFFRNTFDDQRKLVKATAAFERLSIDFKGPVPTNNNNKFILTVIDEFSRFPFAFPCSDVSSK 976

+KPRFFRNTFDDQRKLVKATAAFERLSIDFKGPVPTNNNNKFILTVIDEFSRFPFAFPCSDVSSK

Gypsy-32_BomMor 897 IKPRFFRNTFDDQRKLVKATAAFERLSIDFKGPVPTNNNNKFILTVIDEFSRFPFAFPCSDVSSK 961

Gypsy-6_MelCin 977 TVIKHLNNLFMIFGMPSYVHSDRGTAFLSAEVQEFLHVRGIATSRTTAYNPQGNGQVEKLNSTL1040

TVIKHLNNLFMIFGMPSYVHSDRGTAFLSAEVQEFLHVRGIATSRTTAYNPQGNGQVEKLNSTL

Gypsy-32_BomMor 962 TVIKHLNNLFMIFGMPSYVHSDRGTAFLSAEVQEFLHVRGIATSRTTAYNPQGNGQVEKLNSTL1025

Gypsy-6_MelCin 1041 WRTILLALKTKNLSVEDWEQVLPQALHSIRSLLCTAINCTP 1081

WRTILLALKTK+LSVE+WEQVLPQALHS+RSLLCT INCTP

Gypsy-32_BomMor 1026 WRTILLALKTKHLSVENWEQVLPQALHSVRSLLCTTINCTP 1066

**03. hAT-14_AcaGen#DNA/hAT-Tip100 *vs* hAT-2_NilLug#DNA/hAT-Tip100**

Expect Identities Positives Gaps

4e-46 97/133 (72%) 122/133 (91%) 0/133 (0%)

hAT-14_AcaGen 5 HLNSILEFNLLGKVDIRQQLDSAFRSNVKKHNEQVTKNRYVLSKIIDCILFCGAFELALRGHDER 69

H+++ L+FNLLGK+D+RQQLDSA+R +++KHNEQVTKNRYVLSK+IDCI FCGAFELALRGH E

hAT-2_NilLug 119 HISASLDFNLLGKIDVRQQLDSAYRLSIRKHNEQVTKNRYVLSKLIDCIKFCGAFELALRGHRED 183

hAT-14_AcaGen 70 DDSLNPGVFRGLINFSAELDSSLKDHLTSATVFKGTSKEIQNDLLDCMLTVCQNHIKNEISKASF 134

D++LNPG+F+GL+NF+AELDS+LK+HL SATVFKGTSK+IQN+LLDCML+VCQ +IK EI A +

HAT-2_NilLug 184 DNALNPGIFKGLVNFTAELDSALKEHLNSATVFKGTSKDIQNELLDCMLSVCQENIKQEIKNAQY 248

hAT-14_AcaGen 135 VSV 137

VS+

HAT-2_NilLug 249 VSL 251

**04. Mariner-23N1_LMi#DNA/TcMar-Mariner *vs* Mariner-23_AcaGen#DNA/TcMar-Mariner**

Expect Identities Positives Gaps

9e-42 82/98 (83%) 87/98 (88%) 6/98 (6%)

Mariner-23N1_LMi 155 *VLVYDCDGVILTHYVPPRQTVNAQYYCSFLEHHLRPALRKKRQHFLRNPPIILHDNARAHTAQA 219

* LVYDCDGVILTH VP QTVNAQYY FLEH+LR ALRKKR+HFL+NPPIILHDNAR+H

Mariner-23_AcaGen 186 *SLVYDCDGVILTHAVPTGQTVNAQYYAHFLEHNLRAALRKKRRHFLKNPPIILHDNARSH---- 246

Mariner-23N1_LMi 220 VAALFGRWDWEVLYHPPYSPDLSPCDFDLIPKMKEPLRG 258

V AL RW WEVLYHPPYSP +SPCDFDL PKMKEPLRG

Mariner-23_AcaGen 247 VLALC-RWGWEVLYHPPYSP-MSPCDFDLFPKMKEPLRG 283

**05. BOTMAR1#DNA/TcMar-Mariner *vs* Mariner-9_MesMar#DNA/TcMar-Mariner**

Expect Identities Positives Gaps

1e-41 94/113 (83%) 105/113 (92%) 0/113 (0%)

BOTMAR1 9 NFVPGNYDLRTALIFCYHLKKTAAESHRMLVEAYGEHALGKSQCFEWFKKFRSGNFDARNEERGR 73

+F +YDLRT+LIFCYHLKKTAAESHRMLVEAYGEHALGKSQC+EWF KF+SG+FD RNEERGR

Mariner-9_MesMar 18 DFCAYSYDLRTSLIFCYHLKKTAAESHRMLVEAYGEHALGKSQCYEWFNKFKSGDFDVRNEERGR 82

BOTMAR1 74 PPKKFRDSELQASLDEDDAQTQQQLADQLNVTREAVSIRLKAMGRSRR 121

PPKKF DSELQA LDEDDAQTQ+QLADQLNV+REAVSIRLKAMG+ ++

Mariner-9_MesMar 83 PPKKFEDSELQALLDEDDAQTQKQLADQLNVSREAVSIRLKAMGKIQK 130

**06. MAR1_BM#DNA/TcMar-Tc1 *vs* Mariner-4_SpoFru#DNA/TcMar-Tc1**

Expect Identities Positives Gaps

0 315/326 (96%) 319/326 (97%) 0/326 (0%)

MAR1_BM 1 MEWGDKENRIAVIALHKVGMEPNAIFKTLHTLGISKMFVYRAINRCNETSSVCDRKRSGRPRSVR 65

MEWGDKENRIAVIALHKVGMEPNAIFKTLHTLGISKMFVYRAINR NETSSVCDRKRSGRPRSVR

Mariner-4_SpoFru 1 MEWGDKENRIAVIALHKVGMEPNAIFKTLHTLGISKMFVYRAINRYNETSSVCDRKRSGRPRSVR 65

MAR1_BM 66 TKKVVKAVRERIRRNPVRKQKILSREMKIAPRTMSRILKDDLGLAAYKRRTGHFLTDNLKENRVV 130

TKKVVKAVRERIRRNPVRKQKILSREMKIAPRTMSRILKDDLGLAAYKRRTGHFLTDNLK+NRVV

Mariner-4_SpoFru 66 TKKVVKAVRERIRRNPVRKQKILSREMKIAPRTMSRILKDDLGLAAYKRRTGHFLTDNLKKNRVV 130

MAR1_BM 131 KSKQLLKRYAKGGHRKFLFTDEKFFTIEQHFNKQNDRIYAQSSKEASQLVDRVQRGHYPTSVMVW 195

KSKQLLKRYAKGGHRK LFTDE FFTIEQHFNKQNDRIYAQSSKEASQL+DRVQRGHYPTSVMVW

Mariner-4_SpoFru 131 KSKQLLKRYAKGGHRKKLFTDEIFFTIEQHFNKQNDRIYAQSSKEASQLIDRVQRGHYPTSVMVW 195

MAR1_BM 196 WGISYEGVTEPYFCEKGIKTSAQVYQDTILEKVVKPLNNTMFNNQEWSFQQDSAPGHKARSTQSW 260

WG+SYEGVTEPYFCEKGIKTSAQVYQDTILEKVVKPLN TMFNNQ WSFQQDSAPGHKARSTQSW

Mariner-4_SpoFru 196 WGVSYEGVTEPYFCEKGIKTSAQVYQDTILEKVVKPLNITMFNNQVWSFQQDSAPGHKARSTQSW 260

MAR1_BM 261 LETNVSDFIRAEDWPSSSPDLNPLDYDLWSVLESTACSKRHDNLESLKQSVRLAVKIFPMERVRA 325

LE NVSDFIRAEDWPSSSPDLNPLDYDLWSVLESTACSKRHDNLESLKQS+RLAVK FPMERVRA

Mariner-4_SpoFru 261 LEGNVSDFIRAEDWPSSSPDLNPLDYDLWSVLESTACSKRHDNLESLKQSIRLAVKNFPMERVRA 325

MAR1_BM 326 S 326

S

Mariner-4_SpoFru 326 S 326

**07. Mariner-58_LMi#DNA/TcMar-Mariner *vs* Mariner-22_MesMar#DNA/TcMar-Mariner**

Expect Identities Positives Gaps

4e-36 78/99 (78%) 90/99 (90%) 10/99 (10%)

Mariner-58_LMi 60 VRSNVKTMIIVFFYVRGIV-HREFVPPGQTVNQHFYLDVLRRLREDVRRKRPELWRSGDWFLHHD 123

VR +KTM+++ G++ H E + +NQHFYL+VLRRLREDVRRKRPELW+SGDWFLHHD

Mariner-22_MesMar 70 VR*VLKTMLVL-----GVLQHLETMK----INQHFYLEVLRRLREDVRRKRPELWQSGDWFLHHD 125

Mariner-58_LMi 124 NAPAHTALRVTHYLASQRWSVVPHAPYSPDLAPCDFFLFPRMKK 167

NAPAHTALRV HYLAS+ W VVPH PYSPDLAPCDFF+FPRMKK

Mariner-22_MesMar 126 NAPAHTALRVIHYLASRGWPVVPHPPYSPDLAPCDFFIFPRMKK 169

**08. Mariner-36_LMi#DNA/TcMar-Tc1 *vs* Mariner-33_MesMar#DNA/TcMar-Tc1**

Expect Identities Positives Gaps

7e-93 183/218 (83%) 195/218 (89%) 3/218 (1%)

Mariner-36_LMi 130 NFANDMLFHDDDDFLDHVVFSDESTFHLSGHVNTHNVRIWGSENPHEMVQMQRDSPKVTVFCAIS 194

++ ML+HDD+DFLD V+FSDESTFHLSGHVNTHNVRIWGS NP+EMVQ+QRDSPK+ VFCAIS

Mariner-33_MesMar 86 SYKCSMLLHDDEDFLDRVIFSDESTFHLSGHVNTHNVRIWGSANPQEMVQLQRDSPKLNVFCAIS 150

Mariner-36_LMi 195 RRKVYGPFFFGEPTVTGTSYLDTLEQWLFPQLEEDEPENFIFQQDGAPPHWHSEVRDWLNVTVPK 259

RRKVYGPFFFGE TVTG SYLD LE WLFPQLEE EPENFI+QQDGAPPHWH+ VRDWLNV VP+

Mariner-33_MesMar 151 RRKVYGPFFFGEATVTGVSYLDALELWLFPQLEEAEPENFIWQQDGAPPHWHNSVRDWLNVVVPE 215

Mariner-36_LMi 260 RWIGRKGPNDRACFAWPPRSPDLTPCDFFLWGFIKDRVYVPPLPADLPELRNRIEAAVATITEDT 324

RWIGR GPNDRACFAWPPRSPDLTPCDF+LWG K VYVPPLPADLPEL NRIEA VATIT DT

Mariner-33_MesMar 216 RWIGR-GPNDRACFAWPPRSPDLTPCDFYLWGIRKGYVYVPPLPADLPEL-NRIEAVVATITPDT 278

Mariner-36_LMi 325 LINVWEELGYRLDVCRVTNGAHIEHL 350

LI WEEL YRLDVCRVT G HIEHL

Mariner-33_MesMar 279 LIK-WEELAYRLDVCRVTKGVHIEHL 303
