# Supplementary material for: Diversification of Transposable Elements in Arthropods and Its Impact on Genome Evolution
Source: Genes (Basel). 2019 May 6;10(5):338. doi: 10.3390/genes10050338 (PMC6562904; doi:10.3390/genes10050338)
Supplement: Supplementary file 1 [file genes-10-00338-s001.zip › genes-456725-supplementary/genes-456725-SOM.docx]

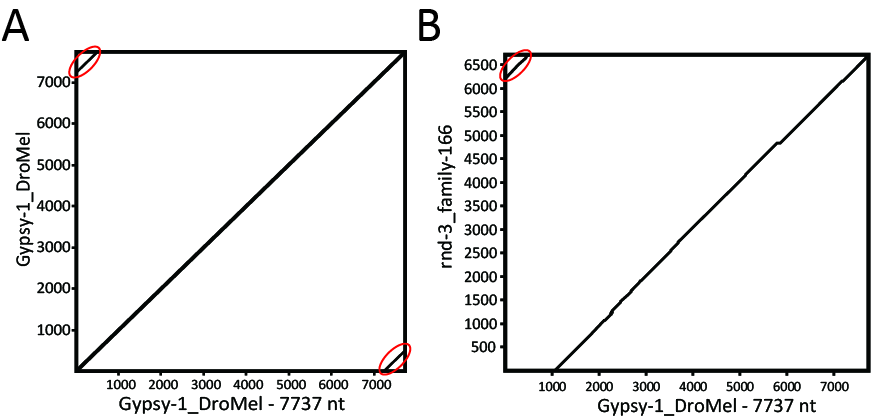


**Figure S1.** The comparison of one TE identified by LTR-specific tools and RepeatModeler. (A) Self-alignment of the full-length *Gypsy-1_DroMel* that was identified by both LTR_Finder and LTRharvest. (B) RepeatModeler only identified a partial sequence of *Gypsy-1_DroMel* (called *rnd-3_family-166* in the RepeatModeler results, *y-axis*) relative to the full-length *Gypsy-1_DroMel* sequence (*x-axis*). The line highlighted by the red circle suggests that the left long terminal repeat had not been identified by RepeatModeler.

**Figure S2.** Sequence divergence distribution of TEs in three *Lepidoptera* species. (A-C) The *y-axis* shows the percentage of the host genomes that is annotated as TEs. The *x-axis* shows Kimura 2-Parameter sequence divergence between individual TE copies and consensus references. Unknown, unclassified TEs; Other, Retro and Retroposon.

.

**Figure S3.** Rate shifts of TE loads in the phylogeny. Red circle: rate shift event; Colors filled in branches are scaled by the evolutionary rates, and rate increases from the cold color (blue) to warm color (red).


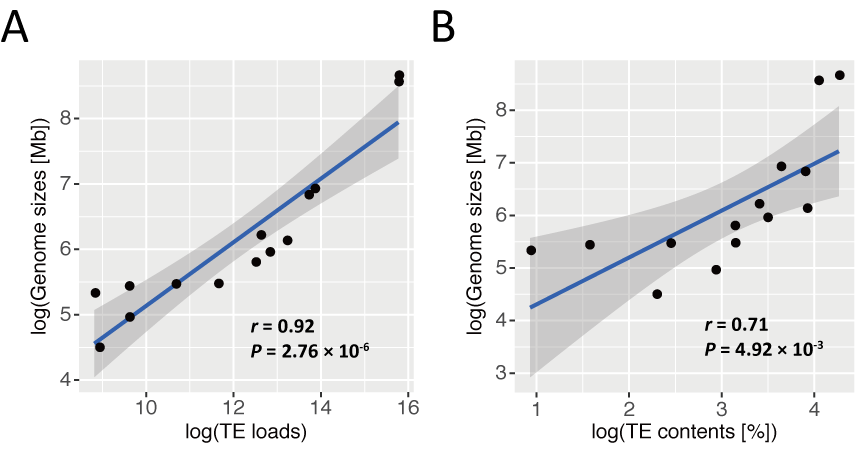


**Figure S4.** The correlation between TEs and host genomes sizes. (A) Linear-regression between TE loads and genomes sizes in natural log(Ln)grams. (B) Linear-regression between TE contents and genomes sizes in natural log(Ln)grams. Each dot is for one species, and the blue line and grey area are the linear regression and the confidence interval. *r* is the Pearson’s product-moment correlation coefficient.

**Figure S5.** The copy numbers of six HTTs in 140 arthropods. (A) The HTT of *Mainer* between *B. terrestris* and *M. martensii*. (B) The HTT of *Gypsy* between *B. mori* and *A. pisum*. (C) The HTT of *Mainer* between *L. migratoria* and *M. martensii*. (D) The HTT of *Mainer* between *L. migratoria* and *M. martensii*. (E) The HTT of *hAT* between *A. geniculata* and *N. lugens*. (F) The HTT of *Mainer* between *L. migratoria* and *A. geniculata*. The species names of hosts of HTT are in bold.

**
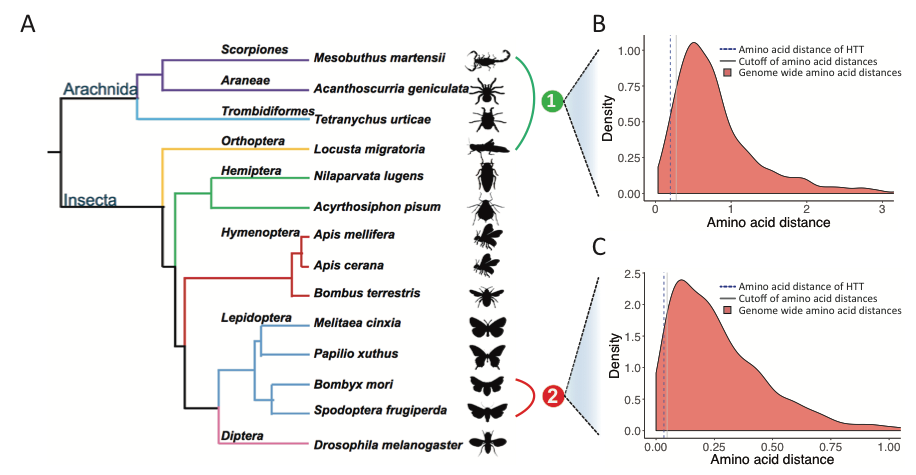
Figure S6.** Two HTTs in arthropods. (A) Phylogeny of the selected species. (B-C) Distribution of the amino acid distances of two HTTs and single-copy orthologous genes. The red density plots show distribution of genome wide amino acid distance. Blue and gray lines indicate the amino acid distance of HTTs and the cutoff for HTT identification respectively.

**Table S1. Basic information about the fourteen species.**

| **Class** | **Order** | **Species** | **Genome**  **sizes** | **Gaps** | **TE content**^†^ | **Genome**  **information** | **Is current genome identical to original publication?** | **References** |
| --- | --- | --- | --- | --- | --- | --- | --- | --- |
| *Insecta* | *Diptera* | *Drosophila melanogaster* | 144 Mb | 0.80% | 21.46% | dmel-6.06 | No | [1] |
|  | *Lepidoptera* | *Spodoptera frugiperda* | 358 Mb | 7.6% | 23.98% | GCA_000753635.2 | Yes | [2] |
|  |  | *Bombyx mori* | 460 Mb | 0.1% | 51.26% | SilkBase (Jan. 2017) | No | - |
|  |  | *Papilio xuthus* | 244 Mb | 2.4% | 24.39% | GCF_000836235.1 | No | [3] |
|  |  | *Melitaea cinxia* | 390 Mb | 1.1% | 34.39% | GCA_000716385.1 | Yes | [4] |
|  | *Hymenoptera* | *Bombus terrestris* | 249 Mb | 4.9% | 13.50% | GCF_000214255.1 | Yes | [5] |
|  |  | *Apis cerana* | 228 Mb | 9.8% | 7.35% | GCA_001442555.1 | No | [6] |
|  |  | *Apis mellifera* | 250 Mb | 8.4% | 10.39% | GCF_000002195.4 | No | [7] |
|  | *Hemiptera* | *Acyrthosiphon pisum* | 542 Mb | 7.7% | 33.53% | GCF_000142985.2 | No | [8] |
|  |  | *Nilaparvata lugens* | 1.14 Gb | 10.8% | 49.98% | GCA_000757685.1 | Yes | [9] |
|  | *Orthoptera* | *Locusta migratoria* | 5.6 Gb | 0 | 67.63% | GCA_000516895.1 | No | [10] |
| *Arachnida* | *Trombidiformes* | *Tetranychus urticae* | 90 Mb | 1.3% | 12.41% | GCF_000239435.1 | Yes | [11] |
|  | *Araneae* | *Acanthoscurria geniculata* | 7.2 Gb | 27.3% | 57.17% | GCA_000661875.1 | No | [12] |
|  | *Scorpiones* | *Mesobuthus martensii* | 925 Mb | 0 | 51.03% | GCA_000484575.1 | No | [13] |

^†^ TE content is defined as the percentage of the sequenced genome that is annotated as TEs (Gaps in the assemblies were excluded).

**Table S2. The weighted small sample size corrected AIC (AW) and 𝝀 of the traits.**

| **Traits** | **AW of comparative models** | | | | **𝝀** | **Number**  **of covered**  **species** | **Kept** |
| --- | --- | --- | --- | --- | --- | --- | --- |
|  | **Brownian**  **motion**  **(BM)** | **Ornstein-**  **Uhlenbeck**  **(OU)** | **Early-**  **burst**  **(EB)** | **White**  **noise** |  |  |  |
| *assembly_length_no_gap* | 0.472 | 0.09 | 0.433 | 0.005 | 1 | 14 | 1 |
| *DNA/Academ* | 0.142 | 0.235 | 0.027 | 0.596 | 0.538 | 7 | 1 |
| *DNA/CMC* | 0.102 | 0.309 | 0.019 | 0.57 | 0.633 | 10 | 1 |
| *DNA/Ginger* | 0.673 | 0.148 | 0.129 | 0.05 | 1 | 7 | 1 |
| *DNA/Harbinger* | 0.071 | 0.312 | 0.014 | 0.603 | 0 | 8 | 1 |
| *DNA/hAT* | 0.642 | 0.196 | 0.123 | 0.039 | 0.952 | 11 | 1 |
| *DNA/PiggyBac* | 0.003 | 0.214 | 0.001 | 0.782 | 0 | 14 | 1 |
| *DNA/TcMar* | 0.139 | 0.223 | 0.027 | 0.611 | 0.854 | 14 | 1 |
| *DNA/*Unclassified | 0.634 | 0.153 | 0.121 | 0.092 | 0.986 | 14 | 1 |
| *LINE/CR1* | 0.617 | 0.234 | 0.118 | 0.031 | 0.951 | 11 | 1 |
| *LINE/Dong-R4* | 0.411 | 0.079 | 0.509 | 0.001 | 1 | 7 | 1 |
| *LINE/I* | 0.264 | 0.307 | 0.05 | 0.379 | 0.801 | 10 | 1 |
| *LINE/Jockey* | 0.005 | 0.311 | 0.001 | 0.683 | 0 | 10 | 1 |
| *LINE/L2* | 0.692 | 0.132 | 0.168 | 0.008 | 1 | 9 | 1 |
| *LINE/R1* | 0.007 | 0.725 | 0.001 | 0.267 | 0.529 | 12 | 1 |
| *LINE/RTE* | 0.371 | 0.343 | 0.071 | 0.215 | 0.863 | 10 | 1 |
| *LINE/*Unclassified | 0.621 | 0.175 | 0.119 | 0.085 | 1 | 14 | 1 |
| *LTR/Copia* | 0.701 | 0.145 | 0.134 | 0.02 | 1 | 14 | 1 |
| *LTR/Gypsy* | 0 | 0.324 | 0 | 0.676 | 0.555 | 13 | 1 |
| *LTR/Pao* | 0.376 | 0.206 | 0.072 | 0.346 | 0.881 | 13 | 1 |
| *LTR/*Unclassified | 0.274 | 0.232 | 0.052 | 0.442 | 0.893 | 13 | 1 |
| *SINE/*Unclassified | 0.711 | 0.136 | 0.145 | 0.008 | 1 | 10 | 1 |
| *sum_DNA* | 0.614 | 0.178 | 0.117 | 0.091 | 0.967 | 14 | 1 |
| *sum_LINE* | 0.571 | 0.23 | 0.109 | 0.09 | 0.945 | 14 | 1 |
| *sum_LTR* | 0.272 | 0.366 | 0.052 | 0.31 | 0.835 | 14 | 1 |
| *sum_SINE* | 0.709 | 0.136 | 0.147 | 0.008 | 1 | 14 | 1 |
| *total_TE* | 0.576 | 0.212 | 0.11 | 0.102 | 0.955 | 14 | 1 |
| *DNA/Crypton* | 0.005 | 0.001 | 0.994 | 0 | 1 | 1 | 0 |
| *DNA/Kolobok* | 0.068 | 0.181 | 0.013 | 0.738 | 0.624 | 3 | 0 |
| *DNA/Kolobok-E* | 0.005 | 0.001 | 0.994 | 0 | 1 | 1 | 0 |
| *DNA/Maverick* | 0 | 0 | 1 | 0 | 1 | 6 | 0 |
| *DNA/Merlin* | 0.614 | 0.174 | 0.117 | 0.095 | 1 | 5 | 0 |
| *DNA/MITE* | 0.007 | 0.257 | 0.001 | 0.735 | 0 | 1 | 0 |
| *DNA/MULE* | 0.514 | 0.216 | 0.098 | 0.172 | 0.903 | 6 | 0 |
| *DNA/P* | 0.286 | 0.055 | 0.657 | 0.002 | 1 | 5 | 0 |
| *DNA/Sola* | 0.273 | 0.228 | 0.052 | 0.447 | 0 | 5 | 0 |
| *DNA/Zator* | 0.066 | 0.302 | 0.013 | 0.619 | 0 | 3 | 0 |
| *LINE/CRE* | 0.241 | 0.499 | 0.046 | 0.214 | 0.49 | 3 | 0 |
| *LINE/CRE-II* | 0.686 | 0.18 | 0.131 | 0.003 | 1 | 4 | 0 |
| *LINE/L1* | 0.002 | 0 | 0.998 | 0 | 1 | 2 | 0 |
| *LINE/LOA* | 0.692 | 0.144 | 0.132 | 0.032 | 1 | 6 | 0 |
| *LINE/Penelope* | 0.69 | 0.14 | 0.132 | 0.038 | 1 | 6 | 0 |
| *LINE/Proto2* | 0.711 | 0.136 | 0.152 | 0.001 | 1 | 4 | 0 |
| *LINE/R2* | 0.001 | 0.163 | 0 | 0.836 | 0 | 6 | 0 |
| *LTR/DIRS* | 0.021 | 0.322 | 0.004 | 0.653 | 0 | 2 | 0 |
| *LTR/Gypsy-Cigr* | 0.488 | 0.197 | 0.093 | 0.222 | 0.346 | 4 | 0 |
| *SINE/I* | 0 | 0 | 1 | 0 | 1 | 1 | 0 |
| *SINE/tRNA* | 0.384 | 0.243 | 0.073 | 0.3 | 0 | 3 | 0 |
| *SINE/tRNA-CR1* | 0.007 | 0.257 | 0.001 | 0.735 | 0 | 1 | 0 |
| *SINE/tRNA-Deu-RTE* | 0 | 0 | 1 | 0 | 1 | 1 | 0 |
| *SINE/tRNA-I* | 0 | 0 | 1 | 0 | 1 | 1 | 0 |
| *SINE/tRNA-RTE* | 0.209 | 0.26 | 0.04 | 0.491 | 0 | 3 | 0 |

Assembly_length_no_gap: the sizes of genomes after excluding Ns.

𝝀: the phylogenetic signal from [14]. 0 < 𝝀 < 1 and greater 𝝀 indicates that the traits could be well predicted by the phylogenetic relationship.

Kept: traits covered by most species (>=7) were used in the next subfamily-level PIC analyses.

Red: best comparative model; blue: acceptable model.

Unclassified: subfamily information is unavailable.

**Table S3. Cutoffs of amino acid distances.**

| **Species pairs** | **Amino acid distances** | | | | **Gene pairs** |
| --- | --- | --- | --- | --- | --- |
|  | **Median** | **Bottom 5%** | **Bottom 10%** | **The 100^th^**  **gene-pair** |  |
| *Acanthoscurria_geniculata~Acyrthosiphon_pisum* | 0.933 | 0.280 | 0.371 | 0.387 | 946 |
| *Acanthoscurria_geniculata~Apis_cerana* | 0.786 | 0.222 | 0.310 | 0.309 | 1,012 |
| *Acanthoscurria_geniculata~Apis_mellifera* | 0.778 | 0.217 | 0.308 | 0.307 | 1,022 |
| *Acanthoscurria_geniculata~Bombus_terrestris* | 0.794 | 0.229 | 0.313 | 0.309 | 1,030 |
| *Acanthoscurria_geniculata~Bombyx_mori* | 0.838 | 0.232 | 0.324 | 0.322 | 1,023 |
| *Acanthoscurria_geniculata~Drosophila_melanogaster* | 0.894 | 0.263 | 0.350 | 0.344 | 1,033 |
| *Acanthoscurria_geniculata~Locusta_migratoria* | 0.714 | 0.205 | 0.277 | 0.331 | 709 |
| *Acanthoscurria_geniculata~Melitaea_cinxia* | 0.911 | 0.257 | 0.350 | 0.373 | 882 |
| *Acanthoscurria_geniculata~Mesobuthus_martensii* | 0.437 | 0.090 | 0.142 | 0.176 | 708 |
| *Acanthoscurria_geniculata~Nilaparvata_lugens* | 0.837 | 0.233 | 0.349 | 0.372 | 855 |
| *Acanthoscurria_geniculata~Papilio_xuthus* | 0.864 | 0.248 | 0.347 | 0.346 | 1,005 |
| *Acanthoscurria_geniculata~Spodoptera_frugiperda* | 0.859 | 0.225 | 0.311 | 0.336 | 884 |
| *Acanthoscurria_geniculata~Tetranychus_urticae* | 0.864 | 0.232 | 0.333 | 0.361 | 847 |
| *Acyrthosiphon_pisum~Apis_cerana* | 0.819 | 0.227 | 0.315 | 0.266 | 1,434 |
| *Acyrthosiphon_pisum~Apis_mellifera* | 0.817 | 0.227 | 0.315 | 0.266 | 1,450 |
| *Acyrthosiphon_pisum~Bombus_terrestris* | 0.827 | 0.228 | 0.329 | 0.266 | 1,463 |
| *Acyrthosiphon_pisum~Bombyx_mori* | 0.872 | 0.253 | 0.352 | 0.301 | 1,445 |
| *Acyrthosiphon_pisum~Drosophila_melanogaster* | 0.910 | 0.275 | 0.373 | 0.314 | 1,466 |
| *Acyrthosiphon_pisum~Locusta_migratoria* | 0.791 | 0.220 | 0.314 | 0.312 | 1,011 |
| *Acyrthosiphon_pisum~Melitaea_cinxia* | 0.954 | 0.273 | 0.367 | 0.338 | 1,232 |
| *Acyrthosiphon_pisum~Mesobuthus_martensii* | 0.863 | 0.246 | 0.354 | 0.362 | 932 |
| *Acyrthosiphon_pisum~Nilaparvata_lugens* | 0.830 | 0.246 | 0.326 | 0.298 | 1,232 |
| *Acyrthosiphon_pisum~Papilio_xuthus* | 0.885 | 0.267 | 0.361 | 0.315 | 1,426 |
| *Acyrthosiphon_pisum~Spodoptera_frugiperda* | 0.875 | 0.253 | 0.345 | 0.321 | 1,241 |
| *Acyrthosiphon_pisum~Tetranychus_urticae* | 1.099 | 0.355 | 0.479 | 0.449 | 1,156 |
| *Apis_cerana~Apis_mellifera* | 0.014 | 0.000 | 0.000 | 0.000 | 1,580 |
| *Apis_cerana~Bombus_terrestris* | 0.097 | 0.008 | 0.017 | 0.011 | 1,588 |
| *Apis_cerana~Bombyx_mori* | 0.701 | 0.155 | 0.255 | 0.201 | 1,568 |
| *Apis_cerana~Drosophila_melanogaster* | 0.765 | 0.209 | 0.306 | 0.234 | 1,592 |
| *Apis_cerana~Locusta_migratoria* | 0.594 | 0.137 | 0.209 | 0.203 | 1,084 |
| *Apis_cerana~Melitaea_cinxia* | 0.745 | 0.185 | 0.273 | 0.241 | 1,328 |
| *Apis_cerana~Mesobuthus_martensii* | 0.697 | 0.182 | 0.283 | 0.282 | 1,009 |
| *Apis_cerana~Nilaparvata_lugens* | 0.697 | 0.158 | 0.259 | 0.220 | 1,332 |
| *Apis_cerana~Papilio_xuthus* | 0.715 | 0.183 | 0.265 | 0.214 | 1,550 |
| *Apis_cerana~Spodoptera_frugiperda* | 0.702 | 0.160 | 0.252 | 0.218 | 1,344 |
| *Apis_cerana~Tetranychus_urticae* | 1.005 | 0.316 | 0.422 | 0.396 | 1,225 |
| *Apis_mellifera~Bombus_terrestris* | 0.098 | 0.009 | 0.016 | 0.011 | 1,600 |
| *Apis_mellifera~Bombyx_mori* | 0.700 | 0.164 | 0.255 | 0.201 | 1,582 |
| *Apis_mellifera~Drosophila_melanogaster* | 0.771 | 0.211 | 0.299 | 0.232 | 1,605 |
| *Apis_mellifera~Locusta_migratoria* | 0.592 | 0.137 | 0.214 | 0.208 | 1,090 |
| *Apis_mellifera~Melitaea_cinxia* | 0.751 | 0.179 | 0.274 | 0.230 | 1,347 |
| *Apis_mellifera~Mesobuthus_martensii* | 0.698 | 0.183 | 0.288 | 0.283 | 1,020 |
| *Apis_mellifera~Nilaparvata_lugens* | 0.696 | 0.181 | 0.261 | 0.221 | 1,343 |
| *Apis_mellifera~Papilio_xuthus* | 0.714 | 0.184 | 0.269 | 0.209 | 1,563 |
| *Apis_mellifera~Spodoptera_frugiperda* | 0.702 | 0.162 | 0.249 | 0.210 | 1,357 |
| *Apis_mellifera~Tetranychus_urticae* | 0.997 | 0.321 | 0.426 | 0.393 | 1,237 |
| *Bombus_terrestris~Bombyx_mori* | 0.714 | 0.171 | 0.265 | 0.208 | 1,596 |
| *Bombus_terrestris~Drosophila_melanogaster* | 0.768 | 0.217 | 0.317 | 0.229 | 1,621 |
| *Bombus_terrestris~Locusta_migratoria* | 0.607 | 0.137 | 0.218 | 0.209 | 1,107 |
| *Bombus_terrestris~Melitaea_cinxia* | 0.759 | 0.187 | 0.284 | 0.240 | 1,356 |
| *Bombus_terrestris~Mesobuthus_martensii* | 0.704 | 0.193 | 0.290 | 0.283 | 1,032 |
| *Bombus_terrestris~Nilaparvata_lugens* | 0.707 | 0.180 | 0.262 | 0.223 | 1,361 |
| *Bombus_terrestris~Papilio_xuthus* | 0.714 | 0.185 | 0.276 | 0.216 | 1,581 |
| *Bombus_terrestris~Spodoptera_frugiperda* | 0.707 | 0.162 | 0.258 | 0.209 | 1,373 |
| *Bombus_terrestris~Tetranychus_urticae* | 1.013 | 0.310 | 0.429 | 0.393 | 1,246 |
| *Bombyx_mori~Drosophila_melanogaster* | 0.760 | 0.203 | 0.305 | 0.235 | 1,600 |
| *Bombyx_mori~Locusta_migratoria* | 0.698 | 0.186 | 0.259 | 0.249 | 1,090 |
| *Bombyx_mori~Melitaea_cinxia* | 0.296 | 0.051 | 0.078 | 0.065 | 1,350 |
| *Bombyx_mori~Mesobuthus_martensii* | 0.769 | 0.208 | 0.284 | 0.277 | 1,023 |
| *Bombyx_mori~Nilaparvata_lugens* | 0.783 | 0.204 | 0.298 | 0.262 | 1,345 |
| *Bombyx_mori~Papilio_xuthus* | 0.249 | 0.044 | 0.070 | 0.053 | 1,568 |
| *Bombyx_mori~Spodoptera_frugiperda* | 0.226 | 0.035 | 0.059 | 0.047 | 1,365 |
| *Bombyx_mori~Tetranychus_urticae* | 1.036 | 0.307 | 0.442 | 0.405 | 1,237 |
| *Drosophila_melanogaster~Locusta_migratoria* | 0.747 | 0.204 | 0.306 | 0.285 | 1,102 |
| *Drosophila_melanogaster~Melitaea_cinxia* | 0.813 | 0.227 | 0.331 | 0.274 | 1,364 |
| *Drosophila_melanogaster~Mesobuthus_martensii* | 0.828 | 0.220 | 0.332 | 0.325 | 1,030 |
| *Drosophila_melanogaster~Nilaparvata_lugens* | 0.847 | 0.231 | 0.336 | 0.270 | 1,360 |
| *Drosophila_melanogaster~Papilio_xuthus* | 0.757 | 0.221 | 0.311 | 0.251 | 1,583 |
| *Drosophila_melanogaster~Spodoptera_frugiperda* | 0.752 | 0.206 | 0.292 | 0.248 | 1,373 |
| *Drosophila_melanogaster~Tetranychus_urticae* | 1.081 | 0.321 | 0.462 | 0.408 | 1,253 |
| *Locusta_migratoria~Melitaea_cinxia* | 0.768 | 0.207 | 0.285 | 0.298 | 920 |
| *Locusta_migratoria~Mesobuthus_martensii* | 0.666 | 0.184 | 0.274 | 0.310 | 719 |
| *Locusta_migratoria~Nilaparvata_lugens* | 0.658 | 0.139 | 0.227 | 0.236 | 919 |
| *Locusta_migratoria~Papilio_xuthus* | 0.708 | 0.201 | 0.271 | 0.264 | 1,079 |
| *Locusta_migratoria~Spodoptera_frugiperda* | 0.695 | 0.184 | 0.254 | 0.260 | 943 |
| *Locusta_migratoria~Tetranychus_urticae* | 0.992 | 0.322 | 0.421 | 0.453 | 866 |
| *Melitaea_cinxia~Mesobuthus_martensii* | 0.817 | 0.229 | 0.320 | 0.352 | 867 |
| *Melitaea_cinxia~Nilaparvata_lugens* | 0.851 | 0.234 | 0.316 | 0.299 | 1,134 |
| *Melitaea_cinxia~Papilio_xuthus* | 0.272 | 0.045 | 0.067 | 0.058 | 1,336 |
| *Melitaea_cinxia~Spodoptera_frugiperda* | 0.283 | 0.037 | 0.065 | 0.057 | 1,196 |
| *Melitaea_cinxia~Tetranychus_urticae* | 1.106 | 0.342 | 0.476 | 0.471 | 1,041 |
| *Mesobuthus_martensii~Nilaparvata_lugens* | 0.769 | 0.221 | 0.305 | 0.331 | 870 |
| *Mesobuthus_martensii~Papilio_xuthus* | 0.778 | 0.223 | 0.324 | 0.324 | 1,000 |
| *Mesobuthus_martensii~Spodoptera_frugiperda* | 0.784 | 0.210 | 0.297 | 0.321 | 882 |
| *Mesobuthus_martensii~Tetranychus_urticae* | 0.788 | 0.218 | 0.317 | 0.346 | 836 |
| *Nilaparvata_lugens~Papilio_xuthus* | 0.796 | 0.221 | 0.307 | 0.266 | 1,323 |
| *Nilaparvata_lugens~Spodoptera_frugiperda* | 0.796 | 0.194 | 0.290 | 0.275 | 1,151 |
| *Nilaparvata_lugens~Tetranychus_urticae* | 1.077 | 0.338 | 0.470 | 0.448 | 1,058 |
| *Papilio_xuthus~Spodoptera_frugiperda* | 0.226 | 0.033 | 0.058 | 0.047 | 1,348 |
| *Papilio_xuthus~Tetranychus_urticae* | 1.055 | 0.324 | 0.459 | 0.424 | 1,217 |
| *Spodoptera_frugiperda~Tetranychus_urticae* | 1.044 | 0.321 | 0.438 | 0.426 | 1,067 |

Bottom 5% and 10%: amino acid distances of single-copy orthologous gene pairs between each two species were sorted in ascending order, and the 5% and 10% cutoffs was defined as the maximum amino acid distances of the first 5% and 10% orthologous pairs.

100^th^: the genome-wide cutoff was defined as the amino acid distance of the 100^th^ gene pair.

**Table S4. The content of TIR in the 14 arthropod genomes**

| **Species** | **TIR (%)** | **Genome Size (log10)** |
| --- | --- | --- |
| *Acanthoscurria geniculata* | 36.95 | 9.8560 |
| *Acyrthosiphon pisum* | 22.25 | 8.7338 |
| *Apis cerana* | 1.32 | 8.3585 |
| *Apis mellifera* | 3.34 | 8.3984 |
| *Bombus terrestris* | 5.85 | 8.3956 |
| *Bombyx mori* | 19.14 | 8.6631 |
| *Drosophila melanogaster* | 2.28 | 8.1575 |
| *Locusta migratoria* | 31.92 | 9.7604 |
| *Melitaea cinxia* | 14.22 | 8.5910 |
| *Mesobuthus martensii* | 31.85 | 8.9664 |
| *Nilaparvata lugens* | 19.71 | 9.0572 |
| *Papilio xuthus* | 7.23 | 8.3872 |
| *Spodoptera frugiperda* | 9.32 | 8.5539 |
| *Tetranychus urticae* | 5.78 | 7.9582 |

* The content of TIR (%) in one arthropod genome is positively correlated (Pearson’s *r* = 0.899, *P* < 0.0001) with the genome size (log10).

**Table S5. TE loads are significantly correlated with genome sizes.**

| **TE subfamilies** | **Correlation coefficient (*r*)** | ***p*** | **Corrected *p*** |
| --- | --- | --- | --- |
| DNA | 0.839 | 3.34E-04 | 8.02E-03 |
| SINE | 0.795 | 1.17E-03 | 2.58E-02 |
| LINE | 0.778 | 1.75E-03 | 3.50E-02 |
| LTR | 0.706 | 7.01E-03 | 1.05E-01 |
| total TE | 0.828 | 4.74E-04 | 1.09E-02 |

**Table S6. Eight horizontally transferred TEs.**

| **Species pair** | **Query** | **Target** | **TE family** | **FASTA alignment** | | | **The 100^th^**  **gene-pair** | **TE amino**  **acid distance** |
| --- | --- | --- | --- | --- | --- | --- | --- | --- |
|  |  |  |  | **identity** | **length** | **E-value** |  |  |
| *A. pisum~B. mori* | *Gypsy-25_AcyPis* | *Gypsy-60_BomMor* | Gypsy | 76.99 | 745 | 0 | 0.301 | 0.29 |
| *B. mori~M. cinxia* | *Gypsy-6_MelCin* | *Gypsy-32_BomMor* | Gypsy | 94.93 | 1,080 | 2.40E-56 | 0.065 | 0.052 |
| *A. geniculata~N. lugens* | *hAT-14_AcaGen* | *hAT-2_NilLug* | hAT-Tip100 | 72.93 | 133 | 3.90E-46 | 0.372 | 0.361 |
| *A. geniculata~L. migratoria* | *Mariner-23N1_LMi* | *Mariner-23_AcaGen* | Mariner | 83.67 | 104 | 1.50E-41 | 0.331 | 0.209 |
| *B. terrestris~M. martensii* | *BOTMAR1* | *Mariner-9_MesMar* | Mariner | 83.19 | 113 | 1.00E-41 | 0.283 | 0.201 |
| *B. mori~S. frugiperda* | *MAR1_BM* | *Mariner-4_SpoFru* | Mariner | 96.63 | 326 | 0 | 0.047 | 0.034 |
| *L. migratoria~M. martensii* | *Mariner-58_LMi* | *Mariner-22_MesMar* | Mariner | 78.79 | 109 | 2.20E-37 | 0.31 | 0.308 |
| *L. migratoria~M. martensii* | *Mariner-36_LMi* | *Mariner-33_MesMar* | Mariner | 83.94 | 221 | 1.10E-90 | 0.31 | 0.196 |

The 100^th^ gene-pair: the genome-wide amino acid distance cutoffs for identifying HTTs.

References

1. Clark, A.G.; Eisen, M.B.; Smith, D.R.; Bergman, C.M.; Oliver, B.; Markow, T.A.; Kaufman, T.C.; Kellis, M.; Gelbart, W.; Iyer, V.N. Evolution of genes and genomes on the drosophila phylogeny. *Nature* **2007**, *450*, 203-218.

2. Ahola, V.; Lehtonen, R.; Somervuo, P.; Salmela, L.; Koskinen, P.; Rastas, P.; Valimaki, N.; Paulin, L.; Kvist, J.; Wahlberg, N.*, et al.* The glanville fritillary genome retains an ancient karyotype and reveals selective chromosomal fusions in lepidoptera. *Nat. Commun.* **2014**, *5*, 4737.

3. Li, X.; Fan, D.; Zhang, W.; Liu, G.; Zhang, L.; Zhao, L.; Fang, X.; Chen, L.; Dong, Y.; Chen, Y.*, et al.* Outbred genome sequencing and crispr/cas9 gene editing in butterflies. *Nat. Commun.* **2015**, *6*, 8212.

4. Kakumani, P.K.; Malhotra, P.; Mukherjee, S.K.; Bhatnagar, R.K. A draft genome assembly of the army worm, spodoptera frugiperda. *Genomics* **2014**, *104*, 134-143.

5. Park, D.; Jung, J.W.; Choi, B.S.; Jayakodi, M.; Lee, J.; Lim, J.; Yu, Y.; Choi, Y.S.; Lee, M.L.; Park, Y.*, et al.* Uncovering the novel characteristics of asian honey bee, apis cerana, by whole genome sequencing. *BMC Genomics* **2015**, *16*, 1.

6. Elsik, C.G.; Worley, K.C.; Bennett, A.K.; Beye, M.; Camara, F.; Childers, C.P.; de Graaf, D.C.; Debyser, G.; Deng, J.; Devreese, B.*, et al.* Finding the missing honey bee genes: Lessons learned from a genome upgrade. *BMC Genomics* **2014**, *15*, 86.

7. Sadd, B.M.; Barribeau, S.M.; Bloch, G.; de Graaf, D.C.; Dearden, P.; Elsik, C.G.; Gadau, J.; Grimmelikhuijzen, C.J.; Hasselmann, M.; Lozier, J.D.*, et al.* The genomes of two key bumblebee species with primitive eusocial organization. *Genome Biol.* **2015**, *16*, 76.

8. Xue, J.; Zhou, X.; Zhang, C.X.; Yu, L.L.; Fan, H.W.; Wang, Z.; Xu, H.J.; Xi, Y.; Zhu, Z.R.; Zhou, W.W.*, et al.* Genomes of the rice pest brown planthopper and its endosymbionts reveal complex complementary contributions for host adaptation. *Genome Biol.* **2014**, *15*, 521.

9. International Aphid Genomics, C. Genome sequence of the pea aphid acyrthosiphon pisum. *PLoS Biol.* **2010**, *8*, e1000313.

10. Wang, X.; Fang, X.; Yang, P.; Jiang, X.; Jiang, F.; Zhao, D.; Li, B.; Cui, F.; Wei, J.; Ma, C.*, et al.* The locust genome provides insight into swarm formation and long-distance flight. *Nat. Commun.* **2014**, *5*, 2957.

11. Sanggaard, K.W.; Bechsgaard, J.S.; Fang, X.; Duan, J.; Dyrlund, T.F.; Gupta, V.; Jiang, X.; Cheng, L.; Fan, D.; Feng, Y.*, et al.* Spider genomes provide insight into composition and evolution of venom and silk. *Nat. Commun.* **2014**, *5*, 3765.

12. Cao, Z.; Yu, Y.; Wu, Y.; Hao, P.; Di, Z.; He, Y.; Chen, Z.; Yang, W.; Shen, Z.; He, X.*, et al.* The genome of mesobuthus martensii reveals a unique adaptation model of arthropods. *Nat. Commun.* **2013**, *4*, 2602.

13. Grbic, M.; Van Leeuwen, T.; Clark, R.M.; Rombauts, S.; Rouze, P.; Grbic, V.; Osborne, E.J.; Dermauw, W.; Ngoc, P.C.; Ortego, F.*, et al.* The genome of tetranychus urticae reveals herbivorous pest adaptations. *Nature* **2011**, *479*, 487-492.

14. Pagel, M. Detecting correlated evolution on phylogenies - a general-method for the comparative-analysis of discrete characters. *Proc. R. Soc. Lond. B.* **1994**, *255*, 37-45.
